# Characterization of cytokinin signaling and homeostasis gene families in two hardwood tree species: *Populus trichocarpa* and *Prunus persica*

**DOI:** 10.1186/1471-2164-14-885

**Published:** 2013-12-16

**Authors:** Juha Immanen, Kaisa Nieminen, Héctor Duchens Silva, Fernanda Rodríguez Rojas, Lee A Meisel, Herman Silva, Victor A Albert, Torgeir R Hvidsten, Ykä Helariutta

**Affiliations:** 1 Institute of Biotechnology and Department of Biosciences, University of Helsinki, FI-00014 Helsinki, Finland; 2 Finnish Forest Research Institute, Vantaa Research Unit, Jokiniemenkuja 1, FI-01301 Vantaa, Finland; 3 Departamento de Producción Agrícola, Laboratorio de Genómica Funcional & Bioinformática, Universidad de Chile, Facultad de Ciencias Agronómicas, Av. Santa Rosa 11315, 8820808 La Pintana, Santiago, Chile; 4 Universidad Andres Bello, Centro de Biotecnología Vegetal, Facultad de Ciencias Biológicas, República 217, 837-0146 Santiago, Chile; 5 Universidad de Chile, Instituto de Nutrición y Tecnología de los Alimentos (INTA), El Líbano 5524, 7830490 Macul, Santiago, Chile; 6 Department of Biological Sciences, University at Buffalo, Buffalo, NY 14260, USA; 7 Umeå Plant Science Centre, Department of Plant Physiology, Umeå University, 901 87 Umeå, Sweden; 8 Department of Chemistry, Biotechnology and Food Science, Norwegian University of Life Sciences, 1432 Ås, Norway

**Keywords:** Cytokinin signaling, Cytokinin homeostasis, Populus trichocarpa, Black cottonwood, Prunus persica, Peach

## Abstract

**Background:**

Through the diversity of cytokinin regulated processes, this phytohormone has a profound impact on plant growth and development. Cytokinin signaling is involved in the control of apical and lateral meristem activity, branching pattern of the shoot, and leaf senescence. These processes influence several traits, including the stem diameter, shoot architecture, and perennial life cycle, which define the development of woody plants. To facilitate research about the role of cytokinin in regulation of woody plant development, we have identified genes associated with cytokinin signaling and homeostasis pathways from two hardwood tree species.

**Results:**

Taking advantage of the sequenced black cottonwood (*Populus trichocarpa*) and peach (*Prunus persica*) genomes, we have compiled a comprehensive list of genes involved in these pathways. We identified genes belonging to the six families of cytokinin oxidases (CKXs), isopentenyl transferases (IPTs), LONELY GUY genes (LOGs), two-component receptors, histidine containing phosphotransmitters (HPts), and response regulators (RRs). All together 85 *Populus* and 45 *Prunus* genes were identified, and compared to their Arabidopsis orthologs through phylogenetic analyses.

**Conclusions:**

In general, when compared to Arabidopsis, differences in gene family structure were often seen in only one of the two tree species. However, one class of genes associated with cytokinin signal transduction, the CKI1-like family of two-component histidine kinases, was larger in both *Populus* and *Prunus* than in Arabidopsis.

## Background

Cytokinin signaling contributes to the regulation of multiple fundamental processes active in plant development. These include cell division, meristem maintenance, shoot initiation and growth, vascular patterning, flower and seed development, nutrient uptake, chloroplast differentiation and light perception [1-3]. Additionally, this hormone plays a role in regulating several developmental programs defining the life of perennial woody plants, including the activity of vascular cambium, branching pattern of the shoot, and the onset of leaf senescence. The long life span and extensive radial growth contribute to the large size and massive amount of wood present in a tree, creating a stark contrast to the much smaller herbaceous annuals. However, only few studies have thus far been published about the role of cytokinin in the regulation of woody plant development. To facilitate this research, we are now presenting a comprehensive description of cytokinin signaling and homeostasis gene families in two hardwood tree species: *Populus trichocarpa* and *Prunus persica*. Gene identification in tree genomes was based on homology with Arabidopsis genes, as cytokinin homeostasis and signal transduction pathways have been extensively studied and well-characterized in this species [1-3].

Structurally, cytokinins are adenine derivatives; based on side chain identity they can be classified into four groups representing isopentenyladenine (iP), trans-zeatin (tZ), cis*-*zeatin, and aromatic cytokinins. iP and tZ are the bioactive forms of this hormone, to which plants respond through a multistep two-component histidine-aspartate (His-Asp-His-Asp) phosphorelay system [4-6]. The phosphorelay is initiated when a cytokinin ligand binds to a histidine kinase receptor, which triggers autophosphorylation of a His residue. After an intramolecular transfer of the phosphoryl to an Asp residue, it will be transferred to a His in a cytosolic histidine phosphotransfer (HPt) protein. The HPts provide a mobile connection between the cytosol and nucleus; they continuously cycle between these two compartments. In the nucleus, the HPt transfers the phosphoryl onto an Asp in a phospho-accepting response regulator (RRs). RRs can be classified into several different types according to their structure and function. Type-B RRs, which belong to the Myb-transcription factors, activate the transcription of cytokinin primary response genes. Among them are the type-A RRs, which are involved in a negative feedback mechanism that helps to fine-tune the function of cytokinin signaling pathway. Type-A RRs repress activity of type-B RRs [4,7] and are stabilized by HPt mediated phosphorylation (To et al. [8]). Adding further flexibility to the signaling pathway, many of its components are capable of forming both homo- and heterodimers [9-13]. Different combinations of the two-component elements presumably add diversity into the process and outcome of the phosphorelay.

Cytokinin signaling represents an ancient hormonal pathway. All of its components are already present in the genome of moss *Physcomitrella patens*[14,15], indicating that the cytokinin phosphorelay was already functional prior to the development of a well-defined plant vasculature. As compared to the moss, the cytokinin signaling pathway has, however, become more diverse during the evolution of land plants. The number of members in most cytokinin signaling gene families is much higher in the genomes of vascular plants than in *Physcomitrella*[14,15]. In general, the dynamic nature of plant genomes has influenced the evolution of all gene families in vascular plants. All angiosperm lineages have undergone reoccurring genome duplications, indicating that polyploidization confers a fitness advantage for plant species. Each advent of a whole genome duplication is subsequently followed by a gradual gene loss; this rediploidization ultimately promotes a new duplication, allowing the process to repeat in a cyclical manner [16].

To study the structure of cytokinin signaling and homeostasis genes families in woody plants, we sought to characterize and compare them between two hardwood tree species. For the first species in our phylogenetic study, we chose the most common model tree for molecular biology: *Populus trichocarpa*, black cottonwood. *Populus* is a fast growing a dioecious tree, which can reach reproductive maturity in four to six years. *Populus* trees provide a wood source for the pulp and paper industry and have the potential to be developed into a biofuel feedstock [17]. *P. trichocarpa* has a relatively small diploid (2n = 38) genome with the haploid size of 485 Mbp. The first version of genome assembly was published in 2006 by Tuskan et al. [18]. Due to the challenges of genome assembly in a highly heterozygous tree species, only the current, third genome assembly of *P. trichocarpa*, has been able to resolve a large number of reads that were previously published as unassembled scaffolds. Specific loci identities have only recently been assigned to all predicted genes. Thanks to these improvements, we have now for the first time been able to reliably recover a complete set of cytokinin signaling and homeostasis genes from a tree species. Accordingly, we will discuss how our analysis differs from previously published reports of *P. trichocarpa* cytokinin signaling genes [14,19,20].

The second hardwood tree species used in this study is the economically important fruit tree peach, *Prunus persica*. In terms of cultivated surface area, *P. persica* is the third most important temperate fruit crop. Additionally, it is a member of the economically important *Rosaceae* family, which includes important crops such as peaches, apples, pears, cherries, plums, apricots, strawberries, almonds, and roses. An international effort has led to the genome sequencing and development of *Prunus persica* as a genomic model for the *Rosaceae* family [21-23]. This hardwood tree is a self-pollinating diploid (2n = 16), with a short juvenile period (2–3 years) and a genome size of 265 Mbp [22,23].

Currently only a little is known about the role of cytokinin signaling in the regulation of tree or fruit development in *Rosaceae*. The available data indicates that cytokinins are important for fruit development: high hormone levels have been measured in growing peach fruits [24]. It has also been demonstrated that exogenous application of cytokinin on sweet cherry fruits significantly increases fruit size and weight [25]. Additionally, cytokinin treated fruits showed increased fruit firmness, increased fruit soluble solid concentrations and a delay in exocarp coloration [25]. Similar results have also been seen in apples and pears that have been treated exogenously with cytokinin [26-29]. Taking together, these observations indicate that the cytokinin signaling and homeostasis pathways can provide candidate genes for the breeding of fast growing and high quality *Rosaceae* fruits.

The third species chosen for our study is the most common herbaceous model plant, *Arabidopsis thaliana*. Arabidopsis provides an excellent reference genome, as its cytokinin homeostasis and signal transduction pathways have been characterized in detail [1-3]. In addition to the contrast between the woody perennial versus herbaceous annual life cycles, the selected three model species differ in their reproductive strategies. Both *Prunus* and Arabidopsis have hermaphroditic flowers, whereas *Populus* is a dioecious tree whose genomic sequence was derived from a female plant [18].

All three model species belong to the rosid clade of angiosperm plants. *Populus* (*Malpighiales*) and *Prunus* (*Rosales*) belong to the eurosids I subclade (*Fabidae*), whereas Arabidopsis (*Brassicales*) belongs to the eurosids II (*Malvidae*) [30]. They display diverse genome duplication histories: since their last common ancestor, *Populus* lineage has undergone one whole genome duplication, Arabidopis two, and *Prunus* none [18,31,32]. Based on the genome duplication history and number of synonymous nucleotide substitutions, the molecular-clock rate has been calculated to be faster in Arabidopsis than in *Populus*[33]. Due to the genome duplication history and gene evolution rate, the *Populus* genome has on average 1.5 orthologs for each Arabidopsis gene [18], and *Prunus* 0.85 [34] (http://www.rosaceae.org/projects/peach_genome/v1.0/homology). The differences in the cytokinin signaling and homeostasis related gene family sizes are consistent with the general genomic trends. We identified a total of 85 genes from the *Populus trichocarpa* genome and 45 genes from *Prunus persica*, as compared to the 60 Arabidopsis genes. The gene family structures between the two tree species and Arabidopsis were compared through phylogenetic analyses.

## Methods

### Sequence alignments

*Populus* and *Prunus* homologues of Arabidopsis genes were identified by searching the *Populus trichocarpa* genome database versions 1.1 and 3.0 using various bioinformatic tools and databases available via the *Populus* genome portal (http://genome.jgi-psf.org/Poptr1_1/Poptr1_1.home.html; http://www.phytozome.net/search.php), and the *Prunus persica* genome version 1 [23], using databases available via The Genome Portal of the Department of Energy Joint Genome Institute [35] (http://genome.jgi-psf.org/Poptr1_1/Poptr1_1.home.html), and Phytozome portal [36] (http://www.phytozome.net/search.php?org=Org_Ptrichocarpa_v3.0; http://www.phytozome.net/search.php?method=Org_Ppersica). The bioinformatics tools included BLAST searches, Gene Ontology (GO), Kyoto Encyclopedia of Genes and Genomes (KEGG), EuKaryotic Orthologous Groups Database (KOG) and ortholog finder. Arabidopsis sequences were identified using The Arabidopsis Information Resource (TAIR). Amino acid sequences were aligned using ClustalW followed by manual adjustments when needed [37]. The best bidirectional hit (BBH) method was used as the first approach to determine orthologous pairs of the cytokinin signaling and response genes in *Prunus* as described by [38]. Protein sequences were aligned using Jalviewand ClustalW2 [39] followed by manual adjustments where needed. The resulting alignment was precisely back-translated to yield a data matrix of the corresponding nucleotide sequences. Gene models, transcript IDs and physical loci of *Populus* genes used in construction of phylogenetic trees can be found in Additional file 1: Table S1. Gene models and EST support for *Prunu*s genes can be found in Additional file 2: Table S2 (physical loci of the genes are not available in the current 1.0 version of the genome), and TAIR gene numbers of Arabidopsis genes in Additional file 3: Table S3.

### Phylogenetic annotation

We used a maximum likelihood search strategy on amino acid alignments to investigate orthologs and paralogs in the cytokinin signaling and homeostasis gene families. Sequences were aligned using MUSCLE with default settings [40]. A single most optimal tree for each data set was computed using the RaxML BlackBox web server (http://embnet.vital-it.ch/raxml-bb/) running RaxML version 7.2.8 [41]. Default settings were used with the WAG model of molecular evolution including a gamma parameter. One hundred bootstrap samples were generated to assess support for the inferred relationships. Local bootstrap values (in percentages) are indicated for branches with ≥50% support.

## Results and discussion

To characterize the genetic components of cytokinin signaling and homeostasis pathways from *Populus* and *Prunus*, we identified genes belonging to the six families of cytokinin oxidases (CKXs), isopentenyl transferases (IPTs), LONELY GUY genes (LOGs), two-component receptors, histidine containing phosphotransmitters (HPts), and type-B, type-A, and type-C response regulators (RRs). Below we will briefly summarize what is known about each gene family in Arabidopsis, after which we describe them in our two tree species.

### Cytokinin oxidases

**Figure 1:**
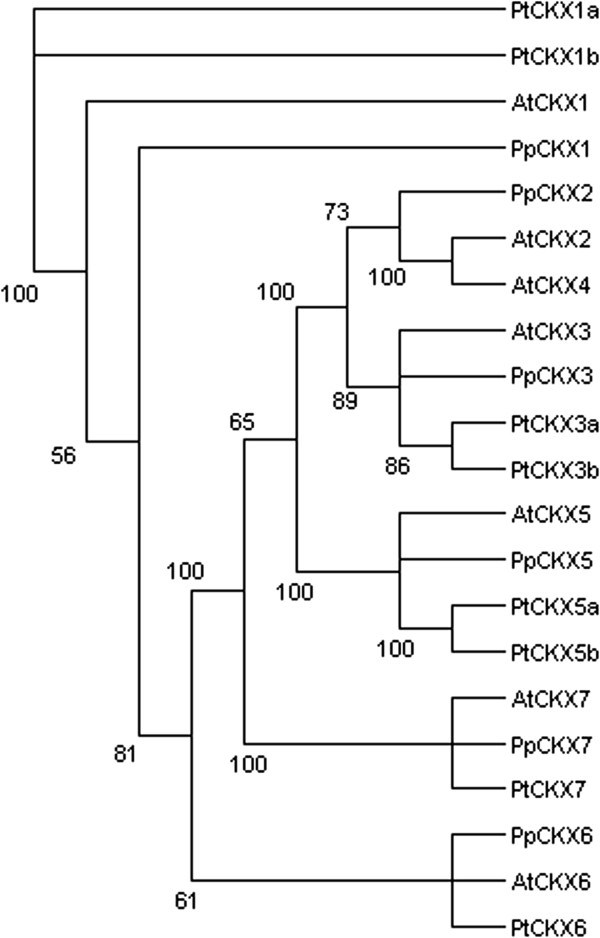
**Unrooted maximum likelihoodtree of *Populus trichocarpa *(Pt), *Prunus persica *(Pp) and Arabidopsis cytokinin oxidase/dehydrogenase (*CKX*) genes coding for enzymes involved in cytokinin catabolism.** The tree is based on a deduced amino acid (431 aa) sequence alignment (Additional file 4: Figure S1). Support for each clade is given as ≥50% of bootstrap pseudoreplicates.

Cytokinin oxidase/dehydrogenases (CKXs) are major enzymes responsible for cytokinin catabolism [42,43]. CKX proteins share low sequence homology; the only conserved features are an oxidoreductase FAD-binding domain and a few short consensus motifs [44]. The *Populus* genome contains eight, *Prunus* six and Arabidopsis seven *CKX* genes (Figure 1, Additional file 4: Figure S1). The structure of the gene family is well conserved between all three species (Figure 1).

### Isopentenyltransferases (IPTs)

**Figure 2:**
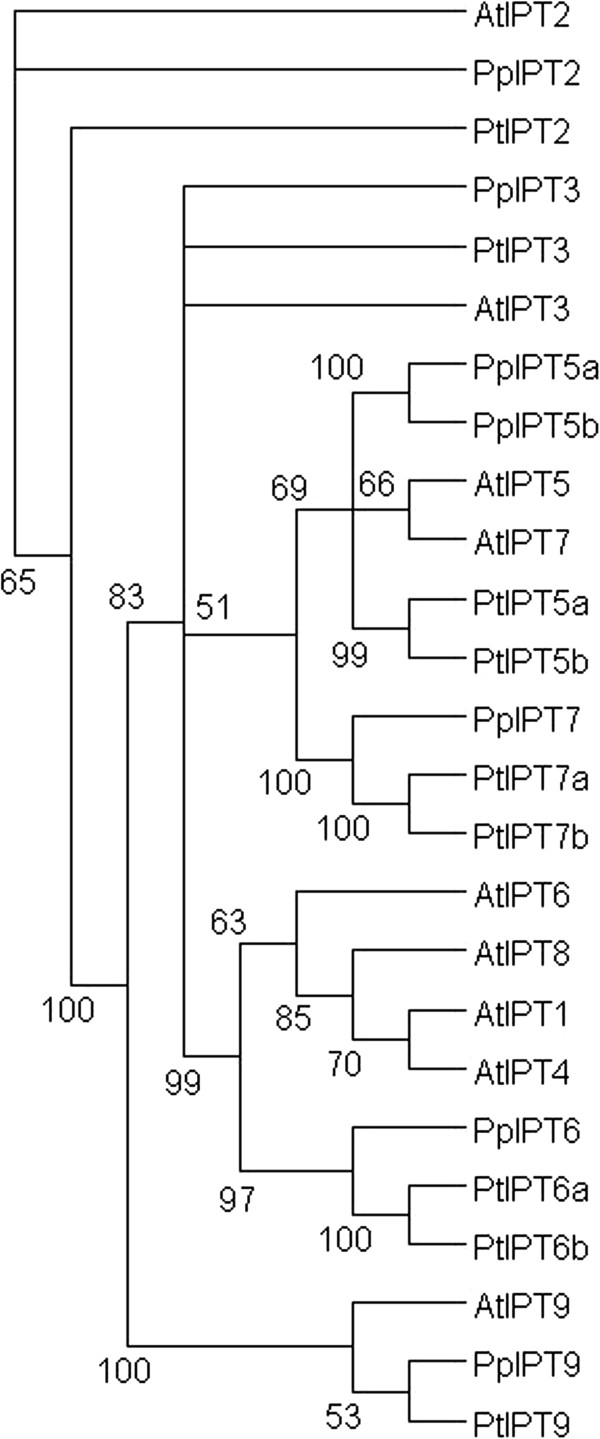
**Unrooted maximum likelihood tree of *Populus *(Pt), *Prunus *(Pp) and Arabidopsis isopentenyl transferase (*IPT*) genes, which encode cytokinin biosynthetic enzymes.** The tree is based on a deduced amino acid (282 aa) sequence alignment (Additional file 5: Figure S2). Support for each clade is given as ≥50% of bootstrap pseudoreplicates.

A major step in cytokinin biosynthesis is catalyzed by ATP/ADP isopentenyltransferases (IPTs), which are responsible for most of the iP and tZ-type cytokinin biosynthesis [45-49]. They belong to the IPT gene family together with tRNA IPTs, which are responsible for the biosynthesis of cZ-type cytokinins [48]. Both *Populus* and Arabidopsis genomes contain nine members of the IPT family, whereas *Prunus* has seven (Figure 2, Additional file 5: Figure S2). Both tree species have one ortholog for each of the two Arabidopsis tRNA IPT genes (*IPT2* and *IPT9*) (Figure 2). The structure of this gene family is otherwise relatively conserved between the three plant species.

### LONELY GUY (LOG) genes

**Figure 3:**
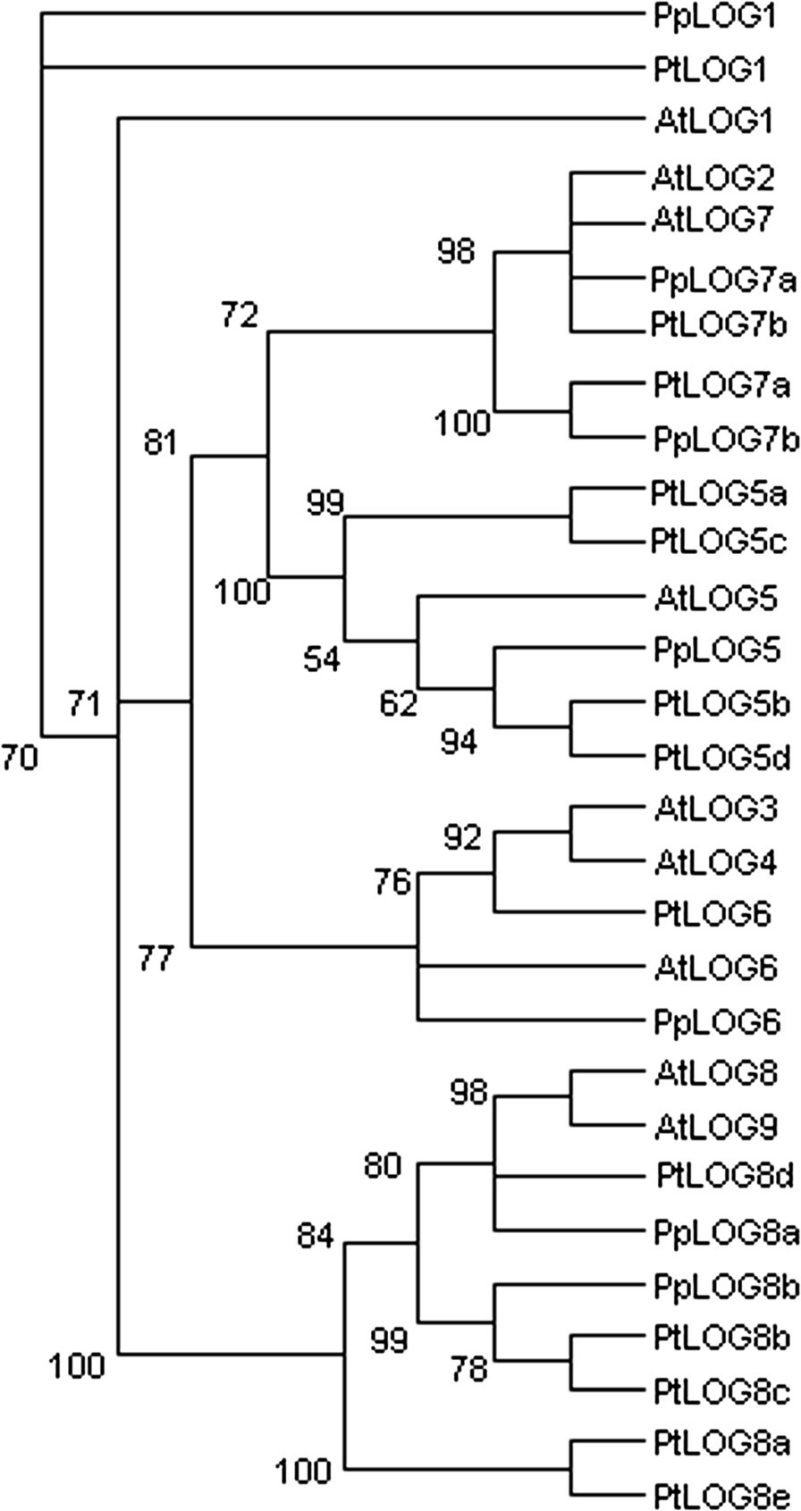
**Unrooted maximum likelihood tree of *Populus *(Pt), *Prunus *(Pp) and Arabidopsis *LONELY GUY *(*LOG*) genes.** LOGs convert conjugated cytokinins into their bioactive forms. The tree is based on a deduced amino acid (198 aa) sequence alignment (Additional file 6: Figure S3). Support for each clade is given as ≥50% of bootstrap pseudoreplicates.

Cytokinin concentration is locally regulated through the activity of LONELY GUY (LOG) enzymes, which convert conjugated cytokinin nucleotides into their bioactive nucleobase forms [50,51]. These enzymes are important regulators of shoot and root apical meristem activity [50-53]. The action of LOGs enables a plant to separate and define the exact site and time of cytokinin activation, and respectively its perception, apart from that of its biosynthesis. The *Populus* genome contains 13 genes coding for cytokinin activating LOG enzymes, *Prunus* has seven and Arabidopsis nine (Figure 3, Additional file 6: Figure S3). The number of orthologs appears to have multiplied in one clade in the *Populus* lineage. This species has four orthologs (*PtLOG5a-d*) of Arabidopis *AtLOG5*, whereas *Prunus* has only one (*PpLOG5*) (Figure 3).

### Two-component receptors

The initial perception of bioactive cytokinins takes place through CRE1-like two-component histidine kinase receptors, which belong to the superfamily of two-component regulators [5,54]. In addition to the cytokinin receptors, this family contains a two-component histidine kinase CKI1 that is able to activate the cytokinin phosphorelay [55], five ethylene receptors (ETR1, ETR2, ERS1, ERS2 and EIN4), five phytochromes (PHYA-E), one putative osmosensor (AtHK1), and a histidine kinase (CKI2/AHK5) associated with ethylene and ABA signaling [56-58]. Our focus will be on the CRE1- and CKI1-like subfamilies that are known to participate in cytokinin signaling phosphorelay.

Arabidopsis has three cytokinin receptors: CRE1/WOL/AHK4, AHK2 and AHK3 [5,54,59]. These receptors have a cytokinin binding CHASE domain, transmembrane domains, a His kinase domain and a receiver domain which contains the phospho-accepting Asp. They share overlapping functions: single null mutants do not have notable phenotypes, whereas the triple mutant is a severely dwarfed and infertile plant [54].

One of the three receptors, CRE1, has both kinase and phosphatase activity: upon binding cytokinin it phosphorylates HPts, whereas in the absence of the hormone it instead dephosphorylates them [6]. Its phosphatase activity helps to quickly inactivate the phosphorelay when the cytokinin levels decrease. In addition to the three canonical receptors, Arabidopsis has a fourth two-component histidine kinase, CKI1, which is capable of inducing cytokinin responses [55]. This kinase can initiate the phosphorelay, but independently of cytokinin [6,60-62]. As it is missing the cytokinin binding CHASE domain, it does not represent a true cytokinin receptor. Further in contrast to the CRE1-like receptors, which are mainly located at the endoplasmic reticulum [11,63], CKI1 appears to be present at the plasma membrane [4,64].

**Figure 4:**
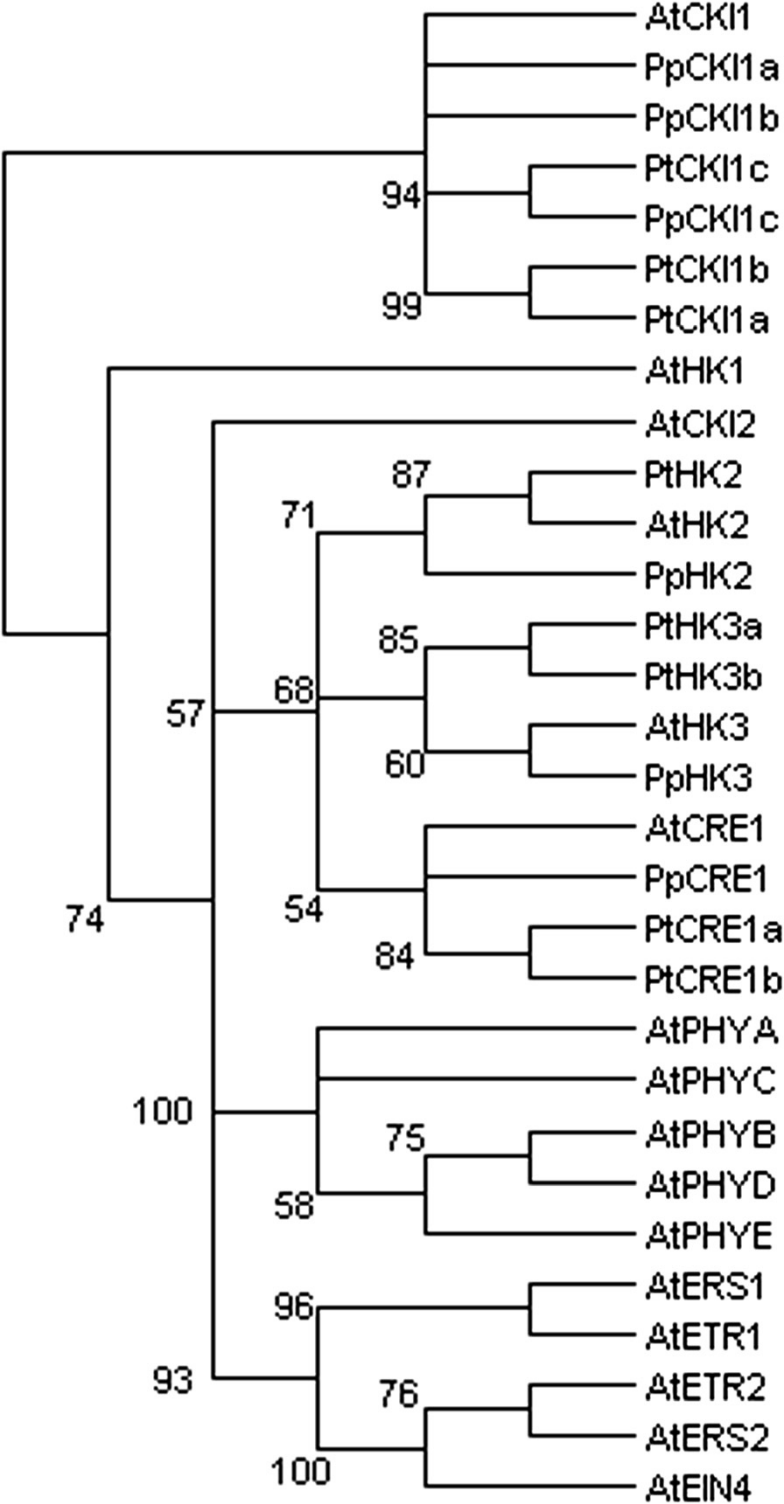
**Unrooted maximum likelihood tree of *Populus *(Pt), *Prunus *(Pp) and Arabidopsis *CRE1*- and *CKI1*-like two-component histidine kinase genes.***CRE1*-like genes encode cytokinin receptors. In contrast, CKI1 does not represent a true receptor: despite being able to activate cytokinin phophorelay, it is unable to bind cytokinin. All the other members of Arabidopsis two-component receptor family, which have no known role in cytokinin signaling, are also included. They include ethylene receptors (ETR1, ETR2, ERS1, ERS2 and EIN4), phytochromes (PHYA-E), a putative osmosensor (AtHK1), and a histidine kinase (CKI2/AHK5) associated with ethylene and ABA signaling. The tree is based on a deduced amino acid (113 aa) sequence alignment (Additional file 7: Figure S4). Support for each clade is given as ≥50% of bootstrap pseudoreplicates.

The *Populus* genome contains five cytokinin receptor genes (*PtCRE1a*, *PtCRE1b*, *PtHK2*, *PtHK3a* and *PtHK3b*) [19], and *Prunus* three (*PpCRE1*, *PpHK2*, *PpHK3*), all orthologous to the three Arabidopsis CRE1-like receptors (Figure 4, Additional file 7: Figure S4). In contrast, both tree species have three orthologs of *CKI1* (*PtCKI1a-c*; *PpCKI1a-c*), a single copy gene in Arabidopsis (Figure 4, Additional file 7: Figure S4). The significant (3-fold) expansion of the *CKI1* gene family appears to be specific for the *Populus* and *Prunus* lineages, as both soybean [65] and rice [66] are similar to Arabidiopsis, having only one ortholog of this gene. CKI1 is known to be essential for female gametophyte development [67], and interestingly, has also been reported to regulate vascular development in Arabidopsis inflorescence stem. In the study by Hejátko et al. [64], *CKI1* expression was detected in vascular tissues, and its over-expression increased the number of vascular cambial cells in vascular bundles. Accordingly, the number of cambial cells was reduced in RNAi lines where the *CKI1* expression level was down-regulated [64]. Therefore, CKI1 appears to have a stimulatory role in regulation of vascular cell proliferation in Arabidopsis.

### Histidine containing phosphotransmitters

Upon binding cytokinin, the CRE1-like receptors initiate phosphorylation of histidine containing phosphotransmitters (HPts), which are continuously cycling between cytosol and nucleus [12]. This movement enables the transfer of phosphoryl groups from the membrane-localized receptors to the nuclear-localized response regulators.

The HPts are characterized by a short motif, HQxKGSSxS, which contains a conserved phospho-accepting His residue (Additional file 8: Figure S5) [68]. In Arabidopsis, five members of the gene family (AHP1-5) contain this canonical consensus motif [69-72]. They share partially redundant functions since higher-order null mutants, that are lacking multiple genes from the same gene family, display a progressively reduced sensitivity to cytokinin [73].

In contrast to the five canonical members, two Arabidopsis HPt genes, *AHP6* and AHP-like (*At4g04402*), contain an atypical motif lacking the conserved His residue [71]. AHP6 has an inhibitory role on the cytokinin phosphorelay, and has been classified as a pseudo HPt [74]. AHP6 negatively interferes with the phosphorelay, potentially by competing with the other AHPs for interaction with phoshorylated receptors. In Arabidopsis roots, the expression of *AHP6* promotes differentiation of protoxylem, the first xylem cell type that forms in a developing vasculature [74]. The negative function of AHP6 contributes to the generation of distinct and well-defined domains of low cytokinin signaling. The function and expression pattern of the AHP-like gene is not known.

**Figure 5:**
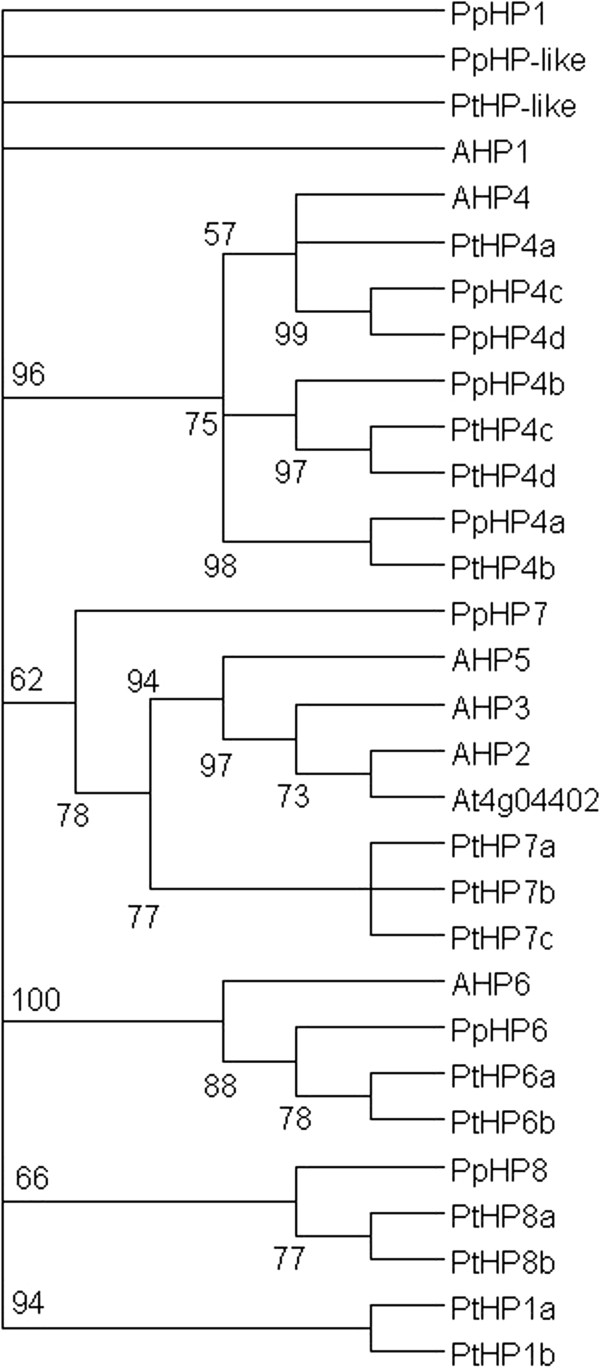
**Unrooted maximum likelihood tree of *Populus *(Pt), *Prunus *(Pp) and Arabidopsis histidine phosphotransfer (*HPt*s) genes.** The tree is based on a deduced amino acid (159 aa) sequence alignment (Additional file 8: Figure S5). Support for each clade is given as ≥50% of bootstrap pseudoreplicates.

All together 14 HPt-encoding genes were identified in the new *Populus* genome assembly (Figure 5, Additional file 8: Figure S5); four more than were reported by Pils and Heyl [14]. Nine *HPts* were identified in *Prunus*, as compared to the seven in Arabidopsis (Figure 5, Additional file 8: Figure S5). The gene family structure is relatively different between the tree species and Arabidopsis (Figure 5). The trees have one clade (*PtHP8a*, *PtHP8b* and *PpHP8*) with no evident Arabidopsis orthologs, and *Populus* has one more (*PtHP1a* and *PtHP1b*) without either an Arabidopsis or *Prunus* ortholog. Two *Populus* (*PtHP6a*, *PtHP6b*) and one *Prunus* HPt (*PpHP6*) are orthologous to the Arabidopsis pseudo HPt *AHP6*, and accordingly lack the phospho-accepting His residue (Additional file 8: Figure S5). One *Populus* (*PtHP-like*) and one *Prunus* gene (*PtHP-like*) contain non-canonical consensus motifs lacking the conserved histidines (Additional file 8: Figure S5); it is not known if these proteins participate in the phosphorelay.

Interestingly, both *Populus* and *Prunus* have four orthologs of a single Arabidopsis gene, *AHP4*. However, this is the case also in two monocot species; rice and maize, which both have three orthologs of this gene [73,75,76]. Compared to other species, it appears that Arabidopsis has lost its *AHP4* homologs during evolution. Somewhat surprisingly, despite the loss of these potentially redundant genes, the phenotype of an Arabidopsis *AHP4* null mutant is not very striking. In Arabidopsis, *AHP4* is primarily expressed in young flowers, and the null mutant shows slightly more secondary cell wall thickening at some anther tissues; yet its fertility is not impaired [77]. Presumably the AHP4 function is still redundant with the other Arabidopsis HPt proteins. One *Populus AHP4* ortholog (*PtHP4b*) is missing the conserved phospho-accepting His residue. This reflects the situation in monocots, where all three rice *AHP4* orthologs [73], and two from the three maize orthologs, also lack the conserved histidine [76]. These genes appear to have evolved into pseudo-response regulators with a potentially inhibitory role on the cytokinin signaling phosphorelay.

### Response regulators

Response regulators (RRs) represent the final components of the cytokinin signaling phosphorelay. The common feature of all RRs is a receiver domain, which contains the phospho-accepting Asp residue as part of the core sequence DD-D-K (Asp Asp-Asp-Lys) [56,57]. The RRs can be classified into four subfamilies: A-type RRs with the receiver domain; B-type RRs with the receiver domain fused to a DNA-binding (GARP) sequence; C-type RRs, which despite an atypical amino acid sequence of their receiver domain, still contain the phospho-accepting Asp residue; and pseudo RRs lacking the conserved Asp in their receiver domain. Members of the type-A, -B and -C RR subfamilies participate in the cytokinin signaling phoshorelay [56,57,78]. The pseudo RRs are, instead of cytokinin signaling, known to function in the regulation of light responses, including circadian rhythms [79-81]. We will not discuss them in this article.

### Type-B RRs

Type-B RRs are DNA-binding transcriptional regulators that positively mediate cytokinin responses [4,82-84]. They activate transcription of cytokinin primary response genes; among them the type-A RRs. The expression of type-B RRs themselves is not induced through cytokinin signaling; their activity is regulated through phosphorylation of a conserved Asp residue in the receiver domain. At least one Arabidopsis B-type RR, ARR2, is rapidly degraded upon its cytokinin induced phophorylation [85]. This mechanism presumably provides proteolysis mediated feedback regulation for its activity. In Arabidopsis, the type-B RRs share partially redundant functions; higher order null mutants show a progressively decreased sensitivity to cytokinin [84,86,87].

**Figure 6:**
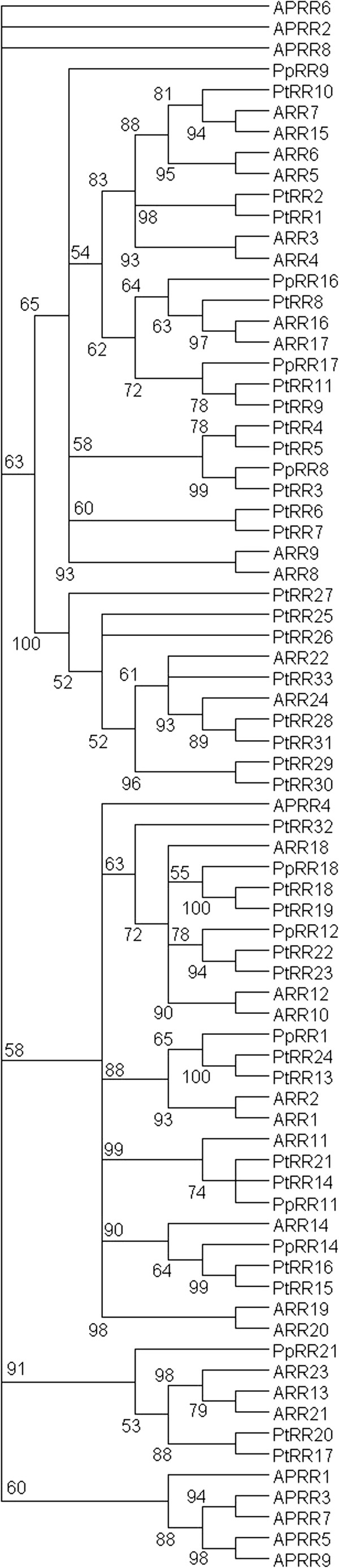
**Unrooted maximum likelihood tree of *Populus *(Pt), *Prunus *(Pp) and Arabidopsis response regulators (RRs).** Members of type-A, -B and –C RRs are involved in cytokinin signaling. For Arabidopsis, also the pseudo response regulator genes with no known role in cytokinin signaling are included. The tree is based on a deduced amino acid (226 aa) sequence alignment for receiver (all RRs) and DNA-binding (GARP) (B-type RRs) domains (Additional file 9: Figure S6). Support for each clade is given as ≥50% of bootstrap pseudoreplicates.

There are six type-B RR genes in *Prunus* genome, whereas *Populus* has thirteen (*PtRR13-25*) and Arabidopsis have both twelve [88] (Figure 6, Additional file 9: Figure S6). Two of the Arabidopsis (*ARR18* and *ARR23*) genes however code for a truncated form of the receiver domain, thus their functionality as RRs is questionable. The structure of *Populus* RR family has previously been reported by Ramírez-Carvajal [20] and Pils and Heyl [14]. In these two reports, altogether 13 type-B *Populus* RRs (*PtRR12-23*) were identified, from which *PtRR12* is missing from the current assembly, whereas *PtRR24* represents a newly identified gene.

### Type-A RRs

The promoters of type-A RR genes contain a high number of B-type RR binding sites [82,83,89,90]. Accordingly, phosphorylated type-B RRs activate the expression of type-A RR genes, which function as negative feedback regulators of cytokinin signaling [8,91-93]. Type-A RRs may repress the type-B RR activity either by competing with them for phosphotransfer from upstream HPts or by forming inactive heterodimers with them [94]. Similar to the B-type, in general, individual A-type RRs are also redundant in Arabidopsis; sensitivity to cytokinin increases progressively in higher order mutants [86,87,91]). Nevertheless, there are also phenotypic differences between Arabidopsis mutants lacking multiple type-A RRs in different combinations. At least two RRs often share highly redundant functions, but these functions differ somewhat from those shared between the other gene pairs [8,91-93]. It seems likely that some functional specificity has evolved between the different RR genes and contributes to their retention in plant genomes.

The *Populus* genome contains eleven types-A RRs (*PtRR1-11*), whereas *Prunus* has only four, compared to the ten genes present in the Arabidopsis (Figure 6, Additional file 9: Figure S6). Both Ramírez-Carvajal et al. [20] and Pils and Heyl [14] identified these same type-A *Populus* RRs (Figure 6). It appears that *Prunus* has lost members of this subfamily during its evolution. However, based on the relatively high redundancy between Arabidopsis RRs, this gene family appears to be well buffered against loss of individual genes.

### Type-C RRs

Type-C, or extra, RRs represent a response regulator subfamily characterized by an atypical receiver domain amino acid sequence [80]. Arabidopsis has two of them, ARR22 and ARR24. They display very restricted expression patterns: *ARR22* is expressed exclusively in developing seeds [95], and *ARR24* only in developing and mature pollen grains [96]. ARR22 can interact and dephosphorylate HPt proteins *in vitro*; it thus appears to represent a negative regulator of the cytokinin signaling phosphorelay [78,95]. In contrast to the type-A RRs, the expression of type-C RRs is not cytokinin inducible [78,96]. Ectopic over-expression of ARR22 gives rise to a distinct phenotype; the plants are dwarf and sterile [78]. Yet surprisingly, neither a vegetative nor a reproductive phenotype was detected in either single or double null mutants of these two genes [95,96]. The function of type-C RRs remains elusive.

Compared to the two Arabidopsis genes, *Populus* has eight type-C RRs (*PtARR26-33*), whereas interestingly, none was found in the *Prunus* genome (Figure 6, Additional file 9: Figure S6). Pils and Heyl [14] identified 10 unnamed type-C *Populus* RRs, four of which have been removed from the new assembly; whereas *PtRR28* and *PtRR33* represent newly identified genes on our list. The expansion of type-C subfamily appears to be specific for the evolution of *Populus* lineage, as we know that several other species have less of them: rice has only two, and both maize and soybean have three [65]. All eight *Populus* type-C RRs appear to share a common ancestor with the two Arabidopsis extra RRs. Two of them, PtRR27 and PtRR29, have an atypical conserved motif (HD-D-K and DD-E-K, respectively), and may represent pseudo response regulators.

## Conclusions

We report here the first comprehensive description of cytokinin signaling and homeostasis gene families in two hardwood tree species; *Populus trichocarpa* and *Prunus persica*. Genomes of both species contain the same cytokinin signal transduction components as Arabidopsis, reflecting the ancient origin of this hormone signaling system. In general, the identified gene families were larger in *Populus* and smaller in *Prunus* when compared to Arabidopsis.

In contrast to the consensus ratios, some cytokinin signaling and homeostasis gene families have distinctively expanded in one or two of the tree species as compared to Arabidopsis. One of the expanded clades is the CKI1-like subfamily of two-component histidine kinases. This family has three members in both *Populus* and *Prunus*, as compared to a single gene in Arabidopsis. This shared expansion indicates that the gene number has probably been multiplied in a common ancestor of the two tree species. Interestingly, in Arabidopsis CKI1 has been shown to participate in the regulation of both reproductive and secondary vascular development. Future research will show if the extra CKI1 orthologs have any role in the control of cambial activity and wood production in tree species.

Another difference is seen in the HPt gene family, where both tree species have four homologs of the single Arabidopsis AHP4 gene. Yet, in this case, several other species also have multiple AHP4 homologs present in their genomes. Some of these homologs appear to represent pseudo HPts, which potentially act to inhibit the cytokinin phosphorelay. It seems that that there has been no tree lineage specific expansion, but that Arabidopsis has instead lost all but one of its AHP4 homologs.

In contrast to the changes shared by both tree species, some gene expansions appear to have taken place only in the *Populus* lineage. One clade of the LOG gene family, the *Populus* orthologs of Arabidopsis *AtLOG5*, has expanded four-fold as compared to either of the two other species. Another gene subfamily, the C-type RRs, has multiplied four-fold in the *Populus* lineage as compared to Arabidopsis, but has instead disappeared from *Prunus*. Possibly other RRs have replaced function of this RR class in *Prunus*. As the function of C-type RRs has remained elusive in Arabidopsis, *Populus* could potentially turn out to be a better model for studying their activity.

We hope that the identification of cytokinin signaling and homeostasis pathway from two hardwood tree species may serve as a reference upon which functional analyses can be developed to determine the role that cytokinin plays in vegetative and reproductive tree development. Additionally, these genes may serve as potential candidate genes for marker-assisted breeding towards increased wood and fruit production.

## Competing interests

The authors declare that they have no competing interests.

## Authors’ contributions

JI and KN participated in the design of the study, in the gene identification from *Populus* and *Prunus* genomes and in the sequence alignment. HDS and FRR participated in the gene identification from *Prunus* genome and in the sequence alignment. VAA performed the phylogenetic analyses. TRH participated in the design of the study. LAM, HS and YH conceived of the study, and participated in its design and coordination. All authors read and approved the final manuscript.

## Supplementary Material

Additional file 1: Table S1 Genetic loci and gene models (transcript IDs) of *Populus trichocarpa* cytokinin signaling and homeostasis genes based on the genome release version 3.0 (http://www.phytozome.net/search.php?org=Org_Ptrichocarpa_v3.0). To enable comparisons with previously published *Populus* gene reports, we have additionally included the respective loci and gene models as they were given in the assembly version 1.1. Click here for file

Additional file 2: Table S2 Gene models and EST support for the *Prunus persica* cytokinin signaling and homeostasis genes. The gene models are given as in the genome release version 1 (http://www.phytozome.net/search.php?method=Org_Ppersica). Click here for file

Additional file 3: Table S3 List of Arabidopsis genes used in the construction of the phylogenetic trees. Click here for file

Additional file 4: Figure S1 Alignment of *Populus trichocarpa* (Pt), *Prunus persica* (Pp) and Arabidopsis cytokinin oxidase/dehydrogenases (CKXs). Click here for file

Additional file 5: Figure S2 Alignment of *Populus* (Pt), *Prunus* (Pp) and Arabidopsis isopentenyl transferases (IPTs). Click here for file

Additional file 6: Figure S3 Alignment of *Populus* (Pt), *Prunus* (Pp) and Arabidopsis LONELY GUY (LOG) proteins). Click here for file

Additional file 7: Figure S4 Alignment of *Populus* (Pt), *Prunus* (Pp) and Arabidopsis CRE1- and CKI1-like two-component histidine kinase, together with Arabidopsis ethylene receptors (ETR1, ETR2, ERS1, ERS2 and EIN4), phytochromes (PHYA-E), a putative osmosensor (AtHK1), and the histidine kinase CKI2/AHK5. Click here for file

Additional file 8: Figure S5 Alignment of *Populus* (Pt), *Prunus* (Pp) and Arabidopsis histidine phosphotransfer proteins (HPts). The consensus HQxKGSSxS motif, containing the phospho-accepting histidine residue (H), is marked above the alignment. Altogether four *Populus* (PtHPt6a, PtHP6b, PHP4b, and PtHP-like), and two *Prunus* (PpHP6 and PpHP-like) HPts lack the conserved histidine residue. Click here for file

Additional file 9: Figure S6 Alignment of *Populus* (Pt), *Prunus* (Pp) and Arabidopsis response regulators (RRs). Click here for file
