# Supplementary material for: Characterization of cytokinin signaling and homeostasis gene families in two hardwood tree species: Populus trichocarpa and Prunus persica
Source: BMC Genomics. 2013 Dec 16;14:885. doi: 10.1186/1471-2164-14-885 (PMC3866579; doi:10.1186/1471-2164-14-885)
Supplement: Additional file 1: Table S1 — Genetic loci and gene models (transcript IDs) of Populus trichocarpa cytokinin signaling and homeostasis genes based on the genome release version 3.0 (http://www.phytozome.net/search.php?org=Org_Ptrichocarpa_v3.0). To enable comparisons with previously published Populus gene reports, we have additionally included the respective loci and gene models as they were given in the assembly version 1.1. [file 1471-2164-14-885-S1.docx]

**Supplementary Table 1**

| **Populus tricho-carpa Gene name** | **assembly 3.0 locus** | **assembly 3.0 transcript ID** | **assembly 1.1 locus** | **gene model *Populus trichocarpa (v1.1)*** |
| --- | --- | --- | --- | --- |
| **PtCKX1a** | **Potri.006G047900** | **POPTR_0006s04650** | LG_VI:3146106-3148350 (-) | fgenesh4_pg.C_LG_VI000395 |
| **PtCKX1b** | **Potri.016G044100** | **POPTR_0016s04340** | LG_XVI:2708599-2710844 (-) | fgenesh4_pg.C_LG_XVI000335 |
| **PtCKX3a** | **Potri.006G152500** | **POPTR_0006s15720** | LG_VI:9177521-9179997 (+) | fgenesh4_pg.C_LG_VI000995 |
| **PtCKX3b** | **Potri.007G066100** | **POPTR_0007s08360** | LG_VII:7246826-7249170 (+) | eugene3.00070818 |
| **PtCKX5a** | **Potri.002G030500** | **POPTR_0002s03190** | LG_II:1973582-1978138 (+) | fgenesh4_pg.C_LG_II000281 |
| **PtCKX5b** | **Potri.005G232300** | **POPTR_0005s25380** | LG_V:16129853-16134528 (-) | fgenesh4_pg.C_LG_V001469 |
| **PtCKX6** | **Potri.003G203600** | **POPTR_0003s20240** | LG_III:17020149-17022322 (-) | fgenesh4_pm.C_LG_III000663 |
| **PtCKX7** | **Potri.006G221000** | **POPTR_0006s23760** | LG_VI:14708339-14712933 (-) | estExt_fgenesh4_pg.C_LG_VI1478 |
|  |  |  |  |  |
| **PtIPT2** | **Potri.009G147600.1** | **POPTR_0009s14980** | LG_IX:1317384-1321337 (-) | fgenesh4_pg.C_LG_IX000210 |
| **PtIPT3** | **Potri.014G139300** | **POPTR_0014s13540** | LG_XIV:6712952-6713923 (-) | gw1.XIV.2886.1 |
| **PtIPT5a** | **Potri.008G202200** | **POPTR_0008s20650** | LG_VIII:14167251-14168234 (-) | fgenesh4_pg.C_LG_VIII001825 |
| **PtIPT5b** | **Potri.010G030500** | **POPTR_0010s03150** | LG_X:3147396-3148379 (+) | fgenesh4_pg.C_LG_X000229 |
| **PtIPT6a** | **Potri.008G121500** | **POPTR_0008s12060** | LG_VIII:7851173-7852261 (+) | eugene3.00081133 |
| **PtIPT6b** | **Potri.010G123900** | **POPTR_0010s13370** | LG_X:12380103-12380935 (-) | eugene3.00101169 |
| **PtIPT7a** | **Potri.004G150900** | **POPTR_0004s15810** | LG_IV:14132299-14133734 (-) | eugene3.00041149 |
| **PtIPT7b** | **Potri.008G033300** | **POPTR_0008s03330** | LG_VIII:1608850-1612009 (+) | eugene3.00080280 |
| **PtIPT9** | **Potri.001G200000** | **POPTR_0001s20710** | LG_I:30947399-30955356 (-) | estExt_fgenesh4_pg.C_LG_I2959 |
|  |  |  |  |  |
| **PtLOG1** | **Potri.009G010800** | **POPTR_0009s01600** | LG_IX:10838415-10841360 (-) | estExt_fgenesh4_pm.C_LG_IX0701 |
| **PtLOG5a** | **Potri.002G012500.1** | **POPTR_0002s01390** | LG_II:721928-723345 (-) | fgenesh4_pg.C_LG_II000113 |
| **PtLOG5b** | **Potri.004G181800** | **POPTR_0004s18850** | LG_IV:16348837-16350267 (+) | estExt_Genewise1_v1.C_LG_IV1427 |
| **PtLOG5c** | **Potri.005G235000.1** | **POPTR_0005s25630** | LG_V:17291847-17293444 (+) | estExt_fgenesh4_pg.C_LG_V1618 |
| **PtLOG5d** | **Potri.009G141500** | **POPTR_0009s14360** | LG_IX:1669489-1670828 (-) | gw1.IX.946.1 |
| **PtLOG6** | **Potri.016G090500** | **POPTR_0016s09180** | LG_XVI:7247514-7251440 (-) | grail3.0004045001 |
| **PtLOG7a** | **Potri.005G248900.1** | **POPTR_0005s27030** | LG_V:16579304-16581729 (+) | estExt_Genewise1_v1.C_LG_V1708 |
| **PtLOG7b** | **Potri.006G204800** | **POPTR_0006s22120** | LG_VI:13480581-13485816 (+) | grail3.0013000402 |
| **PtLOG8a** | **Potri.001G265300.1** | **POPTR_0001s27250** | LG_I:19217356-19218691 | gw1.I.1779.1 |
| **PtLOG8b** | **Potri.001G005400** | **POPTR_0001s04260** | LG_I:364910-367892 (+) | estExt_Genewise1_v1.C_LG_I6442 |
| **PtLOG8c** | **Potri.003G219300.1** | **POPTR_0003s21160** | LG_III:18648710-18651393 (+) | estExt_Genewise1_v1.C_LG_III0865 |
| **PtLOG8d** | **Potri.006G127400** | **POPTR_0006s12920** | LG_VI:15133676-15136449 (-) | estExt_fgenesh4_pg.C_LG_VI1524 |
| **PtLOG8e** | **Potri.009G060300** | **POPTR_0009s06510** | LG_IX:6699503-6700881 | gw1.IX.3326.1 |
|  |  |  |  |  |
| **PtHK2** | **Potri.014G164700.1** | **POPTR_0014s16260** | LG_XIV:9078725-9086099 (-) | fgenesh4_pg.C_LG_XIV001045 |
| **PtHK3a** | **Potri.001G057400.1** | **POPTR_0001s13740** | LG_I:4136702-4142085 | fgenesh4_pm.C_LG_I000182/ |
|  |  |  |  | estExt_fgenesh4_pm.C_LG_I0178 |
| **PtHK3b** | **Potri.003G171000.1** | **POPTR_0003s16950** | LG_III:15230285-15236198 (-) | eugene3.00031406 |
| **PtCRE1a** | **Potri.008G137900.1** | **POPTR_0008s13720** | LG_VIII:9087094-9094028 (-) | fgenesh4_pg.C_LG_VIII001227 |
| **PtCRE1b** | **Potri.010G102900** | **POPTR_0010s11350** | LG_X:10802397-10809431 (+) | fgenesh4_pg.C_LG_X000944 |
| **PtCKI1a** | **Potri.008G191100** | **POPTR_0008s19540** | LG_VIII:13130301-13135074 | gw1.VIII.2896.1/ |
|  |  |  |  | fgenesh4_pg.C_LG_VIII001714 |
| **PtCKI1b** | **Potri.013G009700** | **POPTR_0013s01100** | LG_XIII:701761-705737 (-) | eugene3.00130087 |
| **PtCKI1c** | **Potri.014G121500** | **POPTR_0014s11670** | LG_XIV:5304583-5309313 | gw1.XIV.2340.1/ |
|  |  |  |  | fgenesh4_pg.C_LG_XIV000620 |
|  |  |  |  |  |
| **PtHP1a** | **Potri.008G197600.1** | **POPTR_0008s20220** | LG_VIII:13667870-13669577 (-) | eugene3.00081871 |
| **PtHP1b** | **Potri.010G027100.1** | **POPTR_0010s02780** | LG_X:3704081-3705977 (+) | estExt_fgenesh4_pg.C_LG_X0275 |
| **PtHP4a** | **Potri.001G189900** | **POPTR_0001s19080** | LG_I:14256126-14258090 (+) | fgenesh4_pg.C_LG_I001459/ |
|  |  |  |  | gw1.I.396.1 |
| **PtHP4b** | **Potri.001G465000** | **POPTR_0001s46930** | LG_I:34958715-34959691 (+) | fgenesh4_pg.C_LG_I003334 |
| **PtHP4c** | **Potri.006G236300** | **POPTR_0006s25260** | LG_VI:15867926-15870767 (-) | fgenesh4_pg.C_LG_VI001633 |
| **PtHP4d** | **Potri.009G146300** | **N/A** | LG_IX:1383115-1386626 (+) | fgenesh4_pg.C_LG_IX000220 |
| **PtHP6a** | **Potri.001G191900** | **POPTR_0001s19260** | LG_I:14664592-14665978 (+) | estExt_fgenesh4_pg.C_LG_I1468 |
| **PtHP6b** | **Potri.003G032400** | **POPTR_0003s02690** | scaffold_44:469866-470978 (+) | eugene3.00440046 |
| **PtHP7a** | **Potri.006G098200.1** | **POPTR_0006s09930** | LG_VI:6761152-6763505 (-) | estExt_fgenesh4_pm.C_LG_VI0337 |
| **PtHP7b** | **Potri.014G136200.1** | **POPTR_0014s13200** | LG_XIV:6492990-6494594 (-) | fgenesh4_pg.C_LG_XIV000773 |
| **PtHP7c** | **Potri.016G113500.1** | **POPTR_0016s12090** | LG_XVI:10643531-10646112 (-) | estExt_fgenesh4_pg.C_LG_XVI1041 |
| **PtHP8a** | **Potri.013G028300** | **POPTR_0013s02950** | LG_XIII:1895798-1897733 (+) | estExt_Genewise1_v1.C_LG_XIII1173 |
| **PtHP8b** | **Potri.005G040400** | **POPTR_0005s04220** | scaffold_70:1163740-1165398 (-) | estExt_fgenesh4_pm.C_700061 |
| **PtHP-like** | **Potri.018G046800.1** | **POPTR_0018s08270** | LG_XVIII:3604450-3606086 (-) | eugene3.00180254 |
|  |  |  |  |  |
| **PtRR1** | **Potri.008G193000** | **POPTR_0008s19730** | LG_VIII:15158278-15159506 (-) | eugene3.00081986 |
| **PtRR2** | **Potri.008G193000** | **POPTR_0008s19730** | LG_VIII:13278038-13278842 | estExt_fgenesh1_pg_v1.C_LG_VIII0233/ |
|  |  |  |  | gw1.VIII.329.1 |
| **PtRR3** | **Potri.002G082200** | **POPTR_0002s08270** | LG_II:5721514-5724266 (-) | estExt_fgenesh4_pg.C_LG_II0738 |
| **PtRR4** | **Potri.003G197500.1** | **N/A** | LG_III:17347317-17348069 | estExt_fgenesh1_pg_v1.C_LG_III0215/ |
|  |  |  |  | gw1.III.113.1 |
| **PtRR5** | **Potri.001G027000** | **N/A** | LG_I:2060121-2061214 (+) | eugene3.00010260 |
| **PtRR6** | **Potri.006G041100** | **POPTR_0006s03950** | LG_VI:2665007-2666550 (+) | fgenesh4_pm.C_LG_VI000127 |
| **PtRR7** | **Potri.016G038000.1** | **POPTR_0016s03750** | LG_XVI:2308339-2309809 (+) | fgenesh4_pg.C_LG_XVI000285 |
| **PtRR8** | **Potri.019G058900** | **POPTR_0019s08690** | LG_XIX:7831782-7832640 (+) | fgenesh4_pg.C_LG_XIX000616 |
| **PtRR9** | **Potri.013G156900.1** | **POPTR_0013s15280** | LG_XIII:12808598-12810101 (+) | fgenesh4_pg.C_LG_XIII000527 |
| **PtRR10** | **Potri.015G070000.1** | **POPTR_0015s08130** | LG_XV:4991296-4993461 (-) | estExt_fgenesh4_pg.C_LG_XV0454 |
| **PtRR11** | **Potri.019G058900** | **POPTR_0019s08690** | LG_XIX:10741174-10743356 (-) | eugene3.00190915 |
| **PtRR12** | **N/A** | **N/A** | scaffold_77(542536-538989) | eugene3.00770034 |
| **PtRR13** | **Potri.010G001000.1** | **POPTR_0010s00320** | LG_X:108781-112271 | estExt_fgenesh4_pg.C_LG_XIV046/ |
|  |  |  |  | estExt_Genewise1_v1.C_LG_X3573 |
| **PtRR14** | **Potri.008G181000.1** | **POPTR_0008s18130** | LG_VIII:12178061-12181811 (-) | eugene3.00081689 |
| **PtRR15** | **Potri.008G135500.1** | **POPTR_0008s13490** | LG_VIII:8931292-8934810 (+) | gw1.VIII.1097.1 |
| **PtRR16** | **Potri.010G105600** | **POPTR_0010s11600** | LG_X:10949431-10954136 (-) | fgenesh4_pg.C_LG_X000965 |
| **PtRR17** | **Potri.012G133800.1** | **POPTR_0012s13900** | LG_XII:13242948-13247591 (+) | fgenesh4_pg.C_LG_XII001204 |
| **PtRR18** | **Potri.006G262100.1** | **POPTR_0006s27800** | LG_VI:11563959-11566041 | eugene3.00061255/ gw1.VI.371.1 |
| **PtRR19** | **Potri.018G111300** | **POPTR_0018s11960** | LG_XVIII:11090162-11094620 (-) | estExt_fgenesh4_pg.C_LG_XVIII0961 |
| **PtRR20** | **Potri.015G136000** | **POPTR_0015s13860** | LG_XV:9893242-9898648 (-) | eugene3.00151142 |
| **PtRR21** | **Potri.010G053100** | **N/A** | LG_X:6375038-6378172 (-) | gw1.X.5015.1 |
| **PtRR22** | **Potri.018G021300.1** | **POPTR_0018s02330** | LG_XVIII:6075507-6079496 (+) | fgenesh4_pg.C_LG_XVIII000471 |
| **PtRR23** | **Potri.006G188000** | **POPTR_0006s20220** | LG_VI:17818436-17823270 (+) | fgenesh4_pg.C_LG_VI001883 |
| **PtRR24** | **Potri.010G001000.1** | **POPTR_0010s00320** | LG_X:108781-112274 (+) | estExt_fgenesh4_pm.C_LG_X0005 |
| **PtRR25** | **Potri.018G094700** | **POPTR_0018s10280** | LG_XVIII:9708458-9711867 (-) | fgenesh4_pg.C_LG_XVIII000821 |
| **PtRR26** | **Potri.001G050900.1** | **POPTR_0001s14390** | LG_I:3654850-3656268 (+) | eugene3.00010413 |
| **PtRR27** | **Potri.001G051000** | **POPTR_0001s14380** | LG_I:3657864-3659314 (+) | eugene3.00010414 |
| **PtRR28** | **Potri.002G253000** | **POPTR_0002s25430** | LG_II:23382978-23383501 (-) | eugene3.00002577 |
| **PtRR29** | **Potri.003G177300** | **POPTR_0003s17580** | LG_III:15706236-15707421 (-) | fgenesh4_pg.C_LG_III001494 |
| **PtRR30** | **Potri.003G177400** | **POPTR_0003s17590** | LG_III:15708742-15709867 (-) | fgenesh4_pg.C_LG_III001495 |
| **PtRR31** | **Potri.009G108000** | **POPTR_0009s11120** | LG_IX:3579238-3579785 | fgenesh1_pg.C_LG_IX000565/ |
|  |  |  |  | fgenesh4_pm.C_LG_IX000289 |
| **PtRR32** | **Potri.001G050800** | **POPTR_0001s14400** | LG_I:3651431-3652564 (+) | eugene3.00010412 |
| **PtRR33** | **Potri.019G025000** | **POPTR_0019s04170** | LG_XIX:2908904-2910089 (+) | eugene3.00190251 |
