# Supplementary material for: Characterization of cytokinin signaling and homeostasis gene families in two hardwood tree species: Populus trichocarpa and Prunus persica
Source: BMC Genomics. 2013 Dec 16;14:885. doi: 10.1186/1471-2164-14-885 (PMC3866579; doi:10.1186/1471-2164-14-885)
Supplement: Additional file 2: Table S2 — Gene models and EST support for the Prunus persica cytokinin signaling and homeostasis genes. The gene models are given as in the genome release version 1 (http://www.phytozome.net/search.php?method=Org_Ppersica). [file 1471-2164-14-885-S2.docx]

**Supplementary Table 2**

| ***Prunus persica* Gene name** | **gene model *Prunus persica (v1.0)*** | **ESTs** |
| --- | --- | --- |
| **PpCKX1** | ppa024442 | 0 |
| **PpCKX2** | ppa021859 | 0 |
| **PpCKX3** | ppa021417 | 1 |
| **PpCKX5** | ppa003895 | 1 |
| **PpCKX6** | ppa004120 | 0 |
| **PpCKX7** | ppa004276 | 1 |
|  |  |  |
| **PpIPT2** | ppa026650 | 0 |
| **PpIPT3** | ppa023171 | 0 |
| **PpIPT5a** | ppa025177 | 0 |
| **PpIPT5b** | ppa022463 | 0 |
| **PpIPT6** | ppa025556 | 0 |
| **PpIPT7** | ppa015780 | 0 |
| **PpIPT9** | ppa005482 | 0 |
|  |  |  |
| **PpLOG1** | ppa012603 | 3 |
| **PpLOG5** | ppa010459 | 4 |
| **PpLOG6** | ppa025305 | 0 |
| **PpLOG7a** | ppa016229 | 0 |
| **PpLOG7b** | ppa011245 | 1 |
| **PpLOG8a** | ppa011032 | 2 |
| **PpLOG8b** | ppa022354 | 0 |
|  |  |  |
| **PpHK2** | ppa000361 | 0 |
| **PpHK3** | ppa000679 | 5 |
| **PpCRE1** | ppa000804 | 0 |
| **PpCKI1c** | ppa001196 | 1 |
| **PpCKI1b** | ppa021233 | 0 |
| **PpCKI1a** | ppa024810 | 0 |
|  |  |  |
| **PpHP1** | ppa012790 | 3 |
| **PpHP4a** | ppa014983 | 0 |
| **PpHP4b** | ppa022006 | 0 |
| **PpHP4c** | ppa022068 | 0 |
| **PpHP4d** | ppa025778 | 0 |
| **PpHP6** | ppa014798 |  |
| **PpHP7** | ppa011322 | 1 |
| **PpHP8** | ppa012595 | 6 |
| **PpHP-like** | ppa027183 | 0 |
|  |  |  |
| **PpRR1** | ppa002408 | 9 |
| **PpRR2** | ppa010373 | 3 |
| **PpRR3** | ppa011054 | 1 |
| **PpRR4** | ppa002900 | 1 |
| **PpRR5** | ppa018934 | 0 |
| **PpRR6** | ppa024819 | 0 |
| **PpRR7** | ppa002493 | 1 |
| **PpRR8** | ppa017797 | 0 |
| **PpRR9** | ppa021876 | 1 |
| **PpRR10** | ppa023185 | 1 |
