# Supplementary material for: Characterization of cytokinin signaling and homeostasis gene families in two hardwood tree species: Populus trichocarpa and Prunus persica
Source: BMC Genomics. 2013 Dec 16;14:885. doi: 10.1186/1471-2164-14-885 (PMC3866579; doi:10.1186/1471-2164-14-885)
Supplement: Additional file 3: Table S3 — List of Arabidopsis genes used in the construction of the phylogenetic trees. [file 1471-2164-14-885-S3.doc]

**Supplementary Table 3** List of Arabidopsis genes used in the construction of the phylogenetic trees.

|  | **name** | **gene model** |
| --- | --- | --- |
| **CKXs** | ***CKX1*** | AT2G41510 |
|  | ***CKX2*** | AT2G19500 |
|  | ***CKX3*** | AT5G56970 |
|  | ***CKX4*** | AT4G29740 |
|  | ***CKX5*** | AT1G75450 |
|  | ***CKX6*** | AT3G63440 |
|  | ***CKX7*** | AT5G21482 |
| **IPTs** | ***AtIPT1*** | AT1G68460 |
|  | ***AtIPT2*** | AT2G27760 |
|  | ***AtIPT3*** | AT3G63110 |
|  | ***AtIPT4*** | AT4G24650 |
|  | ***AtIPT5*** | AT5G19040 |
|  | ***AtIPT6*** | AT1G25410 |
|  | ***AtIPT7*** | AT3G23630 |
|  | ***AtIPT8*** | AT3G19160 |
|  | ***AtIPT9*** | AT5G20040 |
| **LOGs** | ***LOG1*** | AT2G28305 |
|  | ***LOG2*** | AT2G35990 |
|  | ***LOG3*** | AT2G37210 |
|  | ***LOG4*** | AT3G53450 |
|  | ***LOG5*** | AT4G35190 |
|  | ***LOG6*** | AT5G03270 |
|  | ***LOG7*** | AT5G06300 |
|  | ***LOG8*** | AT5G11950 |
|  | ***LOG9*** | AT5G26140 |
| **two-component receptors** | ***CRE1*** | AT2G01830 |
|  | ***AHK2*** | AT5G35750 |
|  | ***AHK3*** | AT1G27320 |
|  | ***CKI1*** | AT2G47430 |
|  | ***CKI2/AHK5*** | AT5G10720 |
|  | ***AtHK1*** | AT2G17820 |
|  | ***ETR1*** | AT1G66340 |
|  | ***ERS1*** | AT2G40940 |
|  | ***ETR2*** | AT3G23150 |
|  | ***EIN4*** | AT3G04580 |
|  | ***ERS2*** | AT1G04310 |
|  | ***PHYA*** | AT1G09570 |
|  | ***PHYB*** | AT2G18790 |
|  | ***PHYC*** | AT5G35840 |
|  | ***PHYD*** | AT4G16250 |
|  | ***PHYE*** | AT4G18130 |
| **HPts** | ***AHP1*** | AT3G21510 |
|  | ***AHP2*** | AT3G29350 |
|  | ***AHP3*** | AT5G39340 |
|  | ***AHP4*** | AT3G16360 |
|  | ***AHP5*** | AT1G03430 |
|  | ***AHP6*** | AT1G80100 |
|  | ***AHPlike*** | AT4G04402 |
| **type-A RRs** | ***ARR3*** | AT1G59940 |
|  | ***ARR4*** | AT1G10470 |
|  | ***ARR5*** | AT3G48100 |
|  | ***ARR6*** | AT5G62920 |
|  | ***ARR7*** | AT1G19050 |
|  | ***ARR8*** | AT2G41310 |
|  | ***ARR9*** | AT3G57040 |
|  | ***ARR15*** | AT1G74890 |
|  | ***ARR16*** | AT2G40670 |
|  | ***ARR17*** | AT3G56380 |
| **type-B RRs** | ***ARR1*** | AT3G16857 |
|  | ***ARR2*** | AT4G16110 |
|  | ***ARR10*** | AT4G31920 |
|  | ***ARR11*** | AT1G67710 |
|  | ***ARR12*** | AT2G25180 |
|  | ***ARR13*** | AT2G27070 |
|  | ***ARR14*** | AT2G01760 |
|  | ***ARR18*** | AT5G58080 |
|  | ***ARR19*** | AT1G49190 |
|  | ***ARR20*** | AT3G62670 |
|  | ***ARR21*** | AT5G07210 |
|  | ***ARR23*** | AT5G62120 |
| **type-C RRs** | ***ARR22*** | AT3G04280 |
|  | ***ARR24*** | AT5G26594 |
| **pseudo RRs** | ***APRR1*** | AT5G61380 |
|  | ***APRR2*** | AT4G18020 |
|  | ***APRR3*** | AT5G60100 |
|  | ***APRR4*** | AT5G49240 |
|  | ***APRR5*** | AT5G24470 |
|  | ***APRR6*** | AT1G68210 |
|  | ***APRR7*** | AT5G02810 |
|  | ***APRR8*** | AT4G00760 |
|  | ***APRR9*** | AT2G46790 |
