# Supplementary material for: Characterization of cytokinin signaling and homeostasis gene families in two hardwood tree species: Populus trichocarpa and Prunus persica
Source: BMC Genomics. 2013 Dec 16;14:885. doi: 10.1186/1471-2164-14-885 (PMC3866579; doi:10.1186/1471-2164-14-885)
Supplement: Additional file 4: Figure S1 — Alignment of Populus trichocarpa (Pt), Prunus persica (Pp) and Arabidopsis cytokinin oxidase/dehydrogenases (CKXs). [file 1471-2164-14-885-S4.doc]

**Supplementary Figure 1** Alignment of *Populus trichocarpa* (Pt), *Prunus persica* (Pp) and Arabidopsis cytokinin oxidase/dehydrogenases (CKXs).

AtCKX1 76 VAKDFGNRYQLPPLAILHPRSVFDISSMMKHIVHLGSTSNLTVAARGHGHSLQGQALAHQ
PtCKX1a 74 AAKDFGNMYHFLPSAVLHPKSVSDISNTIKHIFKMGSTSQLTVAARGHSHSLQGQAQAHQ
PtCKX1b 74 AAKDFGNRYHFLPLAVLHPNSVSDISNTIKHIFKMGSTSKLTVAARGHSHSLQGQAQAHQ
PpCKX1 75 AAKDFGNTFQSLPLAVLHPKSVSDISSTIKLIFELGSASELTVAARGHGHSLQGQAQTHG
AtCKX2 45 ASHDFGNITTVTPGGVICPSSTADISRLLQYAA-NGKST-FQVAARGQGHSLNGQASVSG
AtCKX3 58 AATDFGHVTKIFPSAVLIPSSVEDITDLIKLSF-DSQLS-FPLAARGHGHSHRGQASAKD
PtCKX3a 57 VSSDYGNIVHENPAAVLYPSSIEDITSLIKFSY-NNYTP-FTVAARGHGHSVGGQAMASN
PtCKX3b 52 ASTDYGNIVHSTPAAVLYPSSIEDIQILVNSSY-NCPIP-FGISVRGNGHSVNGQDMARD
AtCKX4 52 ASHDFGNITDENPGAVLCPSSTTEVARLLRFANGGFSYNKGSVAARGQGHSLRGQASAPG
AtCKX5 50 VSSDFGMLKSPEPLAVLHPSSAEDVARLVRTAY-GSATA-FPVSARGHGHSNGQAAAGRN
PtCKX5a 46 ASLDFGLLTRAEPMAVLHPGSADDIARLVRAAY-ISSHG-FTVSARGHGHSINGQAQTSN
PtCKX5b 46 ASLDFGLISRSEPMAVLHPGSADDIARLVQAAY-LSSQG-FTVSARGHGHSINGQAQTSN
PpCKX5 47 ASKDFGLMTRAEPLAVLHPGSAQDVARLVRAAY-------FTVSARGHGHSINGQAQTNN
AtCKX6 60 ASKDFGNRYQLIPLAVLHPKSVSDIASTIRHIWMMGTHSQLTVAARGRGHSLQGQAQTRH
PtCKX6 47 AAKDFGNRFQLLPLAVLYPKSVSDIATTIRHIWQMGPNSELTVAARGHSHSLQGQAQAHQ
PpCKX6 59 AARDFGNRYQFLPMAVLHPKTVSDIATTIKHIWDMGPHSELTVAARGHGHSLQGQAQTQR
AtCKX7 50 AGRDFGGMNCVKPLAVVRPVGPEDIAGAVKAAL-RSDK--LTVAARGNGHSINGQAMAEG
PtCKX7 47 AGKDFGGMYTCEPLALIRPASADDVARVVRAAY-RSPN--LTVAARGNGHSINGQAMSDR
PpCKX7 45 ASRDFGGMYSLAPLAFIRPSDADDVARKVKEAA-------LTVAARGNGHSINGQAMADR
PpCKX3 39 ASSDYGHILRENPAAVLYPTSIDEISSLIKFS----------IAARGHGHSTMGQAMAPN
PpCKX2 12 TSTDYGHIVQEFPAAVFNPTSPNDIASLILFSN-------FGVAARGQGHSVRGQDLAPD

AtCKX1 GVVIKMESLRSIRIYKGKQPYVDVSGGEIWINILRETLKYGLSPKSWTDYLHLTVGGTLS
PtCKX1a GIVINMESLQGMQVHTGELPYVDASGSELWINILHETLKYGLAPKSWTDYLHLTVGGTLS
PtCKX1b GIVINMESLQGMQIHTGELPYVDASGGDLWINILHETLKYGLAPKSWTDYLHLTVGGTLS
PpCKX1 GLVINMESLK-MQVHIGEQPYVDVSGGELWINILHETLKYGLSPKSWTDYLHLTVGGTLS
AtCKX2 GVIVNMTCITD-VVVKDK-KYADVAAGTLWVDVLKKTAEKGVSPVSWTDYLHITVGGTLS
AtCKX3 GVVRD----RG-IKVSR-TLYVDVDAAWLWIEVLNKTLELGLTPVSWTDYLYLTVGGTLS
PtCKX3a GVVHKNGTGITVSKCPSLGFYADVGGEQLWIDVLHSTMEHGFAPVSWTDYLYLSVGGTLS
PtCKX3b GVVDK----NG-IRVSK-NLFADVGGEQLWIDVLHTTAAQGLSPVSWTDFLYLSVGGTLS
AtCKX4 GVVVNMTCLAM-AAVISAGTYADVAAGTMWVDVLKAAVDRGVSPVTWTDYLYLSVGGTLS
AtCKX5 GVVVEPVR----------PMYVDVWGGELWVDVLKKTLEHGLAPKSWTDYLYLTVGGTLS
PtCKX5a GVVGSRFGLRK---PQVSIKHVDVWGGELWIDVLRSTLEHGLAPKSWTDYLYLSVGGTLS
PtCKX5b GVVGSRLGLGN---PQVAVMHVDVWGGELWIDVLRSTLEHGLAPKSWTDYLYLSVGGTLS
PpCKX5 GRVMSRVRSGS---GRVSEMYVDAWGGELWIDVLRSTLEYGLAPKSWTDYLYLSVGGTLS
AtCKX6 GIVIHMESLHPLQVYSVDSPYVDVSGGELWINILHETLKYGLAPKSWTDYLHLTVGGTLS
PtCKX6 GVVINMESLQGMHVYTGNNPYVDASGGELWIDILRECLEYGLAPKSWTDYLHLTVGGTLS
PpCKX6 GVVINMESLEGIQVYTGSSPYVDVSGGELWINILHESLKYGLAPKSWTDYLHLTIGGTLS
AtCKX7 GLVN-------VGYLSGD-AFVDVSG-ALWEDVLKCVSEYGLAPRSWTDYLGLTVGGTLS
PtCKX7 GLVN-------VVRMNG--TFVDVSG-ALWEDVLKCVLEYKLAPRSWTDYLGLTVGGTLS
PpCKX7 GLVLDMRSLDDVVEAN---FYADVSGGALWEHVLKCVSEYGMAPRSWTDYLSLTVGGTLS
PpCKX3 GVVVDS-HASG-IIVNN-YYYADVGGGQLWIDVLHASLEHGLAPVAWTDYLYLTVGGTLS
PpCKX2 GVVINMTALSN-IVV-----YADVGGEQLWIDVLHATLDHGLTPVSWTDYLYLSVGGTLS

AtCKX1 NAGISGQAFKHGPQINNVYQLEIVTGKGEVVTCSEKRNSELFFSVLGGLGQFGIITRARI
PtCKX1a NAGISGQAFKHGPQINNIYQLEVVTGKGEAVTCSENKNADLFYGVLGGLGQFGIITRARI
PtCKX1b NAGISGQAFKHGPQINNIYQLEVVTGKGEVVTCTEKQNAELFYSVLGGLGQFGIITRARI
PpCKX1 YAGISGQAFRHGPQINNVYQLEVVTGQGEVITCSENKSPDLFYGVLGGLGQFGIITQARI
AtCKX2 NGGIGGQVFRNGPLVSNVLELDVITGKGEMLTCSRQLNPELFYGVLGGLGQFGIITRARI
AtCKX3 NGGISGQTFRYGPQITNVLEMDVITGKGEIATCSKDMNSDLFFAVLGGLGQFGIITRARI
PtCKX3a NAGISGTTFRYGPQISNVYEMDVVTGKGELVTCSSHTNSELFYAVLGGLGQFGIITRARI
PtCKX3b NAGVSGQTFLHGPQISNVYELDVITGKGELVTCSKRNNSDLFDSVLGGLGQFGIITRARI
AtCKX4 NAGIGGQTFRHGPQISNVHELDVITGKGEMMTCSPKLNPELFYGVLGGLGQFGIITRARI
AtCKX5 NAGISGQAFHHGPQISNVLELDVVTGKGEVMRCSEEENTRLFHGVLGGLGQFGIITRARI
PtCKX5a NGGISGQAFNHGPQISNVYELDVVTGKGELSTCSEEKNSELFHAVLGGLGQFGIITRARI
PtCKX5b NGGISGQAFNHGPQISNVYELDVVTGKGELMTCSEEKNSKLFHAVLGGLGQFGIITRARI
PpCKX5 NAGISGQAFNHGPQISSVDELDVVTGRGELLTCSEEKNSELFHAVLGGLGQFGIITRARI
AtCKX6 NAGISGQAFRHGPQISNVHQLEIVTGKGEILNCTKRQNSDLFNGVLGGLGQFGIITRARI
PtCKX6 NAGVSGQAFRHGPQISNVHQMEVVTGKGEVLNCSEKQNSDLFHSVLGGLGQFGIITRARI
PpCKX6 NAGISGQAFRHGPQISNVHQLEVVTGKGEVINCSEEQNEDLFHSVLGGLGQFGIITRARI
AtCKX7 NAGVSGQAFRYGPQTSNVTELDVVTGNGDVVTCSEIENSELFFSVLGGLGQFGIITRARV
PtCKX7 NAGVSGQAFRFGPQTCNVAELDVVTGEGQLMTCNKNENSELFFGALGGLGQFGIVTRARV
PpCKX7 NAGVSGQAFRYGPQTSNVTELQVVTGKGEIFNCSETENSELFFGALGGLGQFGIITRARV
PpCKX3 NAGISGSAFRFGPQISNVYEMDVVTGQGDFVTCSPQNNSDLFFGVLGGLGQFGIITRARI
PpCKX2 NAGISGQTFRFGPQISNVYELDVVTGKGDFITCSSTKKPKLFYAVLGGLGQFGIITRARI

AtCKX1 SLEPAPHMVKWIF--SRDQEYLISK----EKTFDYVEGFVIINRTDLLNNWRS-SFSPND
PtCKX1a SLEPAPKMVKWI--FSSDQEHLIS-----KNSFDYIEGLVIINRTGLLNNWRS-SFNPKD
PtCKX1b SLEPAPKMVKWI--FSNDQERLISS----KDSFDYIEGLVIINRTGLLNNWRS-SFNPKD
PpCKX1 SLEPAPKMVKWIR-FTKDQEFLISS----ENSFDYIEGFVIINRTGLLNNWRS-SFNPKD
AtCKX2 VLDHAPKRAKW---FFTKDQ----ERLIG---VDYLEGQIFLSNGV----VDTSFFPPSD
AtCKX3 KLEVAPKRAKWLRFLYIDFS-----IS-KTDGVDFLEGSIMVDHGPPDNWR-STYYPPSD
PtCKX3a ALEPAPKRVKWVRMLYSDFS---ISIN--KNALDYLEGSLLMAQGPPNNWR-SSFFPSSD
PtCKX3b ALRSAPTKVRWSRAFYSNFS-----VRGGRDVANYLEGSLMLDNGTPTEWI-TSFFHPTQ
AtCKX4 ALDHAPTRVKWSRILYSDFS----AFKRG---VDFLEGQLMMSNGF----VDTSFFPLSD
AtCKX5 SLEPAPQRVRWIRVLYSSFKVFHGQ-----LKFDYVEGFVIVD-EGLVNNWRSSFFSPRN
PtCKX5a ALEPAPQRVRWIRVLYSNFSISHGS-----QKFDYVEGFVIVD-EGLINNWRSSFFSPRN
PtCKX5b ALEPAPQRVRWIRVLYSNFSISHGT-----LKFDYVEGFVIVD-EGLINNWRSSFFSPRN
PpCKX5 ALEPAPQRVRWIRVLYSNFSISHGT-----HKFDYVEGFVIVD-EGLINNWRSSFFSPSN
AtCKX6 ALEPAP---------TMDQEQLISA----GHKFDYIEGFVIINRTGLLNSWRL-SFTAEE
PtCKX6 SLEPAPDMVKWIF--ATDQERLIGA----ENTFDYIEGFVIINRTGLLNNWRS-SFNPQD
PpCKX6 SLEAAPARVKWIR-FTQDQEHLISS----ENTFDYVEGFVIINRTGLLNNWRS-SFNSKD
AtCKX7 LLQPAPDMVRWIRVVYTEFDESQS-------SFDYVEGFVFVNGADPVNGWPTVEFDPTR
PtCKX7 VVQSAPDMVRWIRVVYSEFEDTRE-------SFDYVG-FVFVNSVDPANGWPTVGFDPSR
PpCKX7 LLQPAPDMVRWIRLVYTEFEDFTR------DSFDYVEGFSP--------------FDPTH
PpCKX3 ALEPAPKRVKWVRMLYNDFSAYLISVN-QSNALDYLEGSLLINQGSPNNWR-SSFFPQST
PpCKX2 ALQPAPNRAKWVRLLYSNFS----AFSSG---FDYVEGLLLM-----------SFY----

AtCKX1 STQGK--TLYCLEVVKYFNPEEASSMDQETGKLLSELNYIPSTLFSSEVPYIEFLDRVHI
PtCKX1a PLQG--KTLYCLEIAKYFNPNESDAMNQETESLLSELNYIPSTLFLSEVSYVEFLDRVHL
PtCKX1b PLQG--KTLYCLEIAKYFSPDESDIMNQKTESLLSELSYISSTLFLSEVSYVEFLDRVHL
PpCKX1 PMQAS-RTLYCLEMAKYFNPNETDVMDQRTESLLLLLHYIPSTLFLSEVSYVEFLDRVHL
AtCKX2 QSKHGI-IYVL-EVAKYYDDPNLPIISKVIDTLTKTLSYLPGFISMHDVAYFDFLNRVHV
AtCKX3 HLRIAS-MVKR--VVKYYDETSQYTVNEEMEELSDSLNHVRGFMYEKDVTYMDFLNRVRT
PtCKX3a IPKIMS-LVT--QVAKYYDDGTRHIVDKDLQQLLKGLSFVAGFMFEKDVSFVDFLNRVRS
PtCKX3b LPQIMS-LVKT-LTKYYFIEDIESEKDQDLQQVFKDFSHVPGLINAKFVSYQEFLTRVPN
AtCKX4 QTRVAS-LVND-HRIIYVLETTLPIIDQVIDTLSRTLGFAPGFMFVQDVPYFDFLNRVRN
AtCKX5 PVKISS---VSSNGSKNYHDSDSEIVDQEVEILMKKLNFIPTSVFTTDLQYVDFLDRVHK
PtCKX5a PVKISS---IGANITKNYDEATADTIDQEVEALMKRLNFIPSSVFTTDLPYIDFLDRVHK
PtCKX5b PVKISS---VGANITKNYDESTGDTIDQEVEALMKNLNFIPSTVFTTDLPYTDFLDRVHR
PpCKX5 PVKITS---INSQITKNYHESTADTIDQEIEGLLKKLDFVPTSVFTTDLPYVDFLDRVHK
AtCKX6 PLEGR--TLYCLELAKYLKQDNKDVINQEVKETLSELSYVTSTLFTTEVAYEAFLDRVHV
PtCKX6 PVQGR--TLYCLELAKYFNRDRADALNEEVGNLLSQLRYITSTLFQTEVPYIEFLDRVHV
PpCKX6 PVQAR--TLFCLELAKYFNLDKTDLINEEVENFLSRLSYIRSTLFMSEVAYIDFLDRVHV
AtCKX7 LPQSCG-------SLHYRDSDSNSTIDKRVERLIGRLRFNEGLRFEVDLPYVDFLLRVKR
PtCKX7 VPRTAG-------SVHYQKTDHPSTVDKAVNSLLGRLGFIEDMKFQVDVSYVEFLLRVKH
PpCKX7 LPR------------TYRHADDPSIVDMGVNRLLGGLGFVERLKFQVDLSYLEFLLRVKR
PpCKX3 YSRIIS-LVTKLEVAKYYDQHTETAVDKELELLLKGLSFLPGFVFEKDVAYVEFLNRVKS
PpCKX2 -------------PIKYYDDSTRDTVDKVVEKLTKRLSFVPGFMFEQDVSYEEFLDRVRI

AtCKX1 AERKLRAKGLWEVPHPWLN----KSSIYQFATEVFNNILT-SNNNGPILIYPVNQSKWKK
PtCKX1a SEIKLRAKGLWDIPHPWLN----KNKIFEFAQEVFGNILT-DSSNGPILIYPVNKSKWDN
PtCKX1b SEIKLRSKGLWEIPHPWMN----RTNIIEFAQEVFGNILT-GNSNGPILIYPVNKSKWNN
PpCKX1 SEIKLRTKGLWEVPHPWMNLLIPKSKIHDFADEIFGNILT-DNINGPILMYPVNKTKWNN
AtCKX2 EENKLRSLGLWELPHPWLNK----SRILFHN-----GLLKQKSASG--LLYPTNRNKWDN
AtCKX3 GELNLKSKGQWDVPHPWLNLFVPKTQIDVFK-----GILRNNITSGPVLVYPMNRNKWND
PtCKX3a GEQKLHSQGLWDVPHPWLNLFLPKSRIKVFH-----DVLKRNITTGVVLFYPMNRKKWDD
PtCKX3b AENESQT-------HPWQNLFIPQSRIVVLR-----DVLKRNITTGPVLFYPLNRHKWDA
AtCKX4 EEDKLRSLGLWEVPHPWLNIFVPGSRIQFHD-----GILNQTSTSGVTLFYPTNRNKWNN
AtCKX5 AELKLRSKNLWEVPHPWLNLFVPKSRIS----DVFKGILG-NKTSGPILIYPMNKDKWDE
PtCKX5a AELKLRAKGLWEVPHPWLNLFVPKSRMR----GVFKGILGNNKTSGPILIYPMNKNKWDQ
PtCKX5b AELKLRAKGLWEVPHPWLNLFVPRSRIR----GVFKGILGNNKTSGPILIYPMNKNKWDQ
PpCKX5 AELKLRSKSLWDVPHPWLNLFVPKSRIS----GVFKGILG-NKTSGPILIYPMNKDKWDQ
AtCKX6 SEVKLRSKGQWEVPHPWLN----RSKINEFARGVFGNILT-DTSNGPVIVYPVNKSKWDN
PtCKX6 SEVKLRSKGLWEVPHPWLN----KSKINDFADEVFGNILT-DTSNGPVLIYPVNKSKWDN
PpCKX6 SEIKLRSKDLWDVPHPWLN----KSKIHTFAEEVFGSILT-ATSNGPILIYPVNRSKWDN
AtCKX7 SEEIAKENGTWETPHPWLNLFVSKRDIGD-----VKELVK-NGVNGPMLVYPLLRS--DD
PtCKX7 AEESARENGTWDAPHPWLNMFVSKRDVAD-----FKRMLK-EGVGGPILVYPLLRS--KD
PpCKX7 AEEHAKANGIWDAPHPWLNLFVSKSDIADFDRT--KKILK-DGIGGPMLVYPLLRSKWDA
PpCKX3 GEEKLQSQGQWDVPHPWLNLFVPKSRISDF-------VLKRNITTAPVLLYPMNRTKWDD
PpCKX2 EEKILQSLGLWDIAHPWMNLFVPKSRISDFD----------SGIL-LILIYPMNRNKWDG

AtCKX1 HTSLITPNEDIFYLVAFLPSAVPNSSGKNDLEYLLKQNQRVMNFCAAANLNVKQYLPHYE
PtCKX1a RTSLITPEEDTFYLVAFLSSAMPSSTGRDGLFHILAQNQRILGFCSSTSLGAKQYLPHYS
PtCKX1b RTSLITPDEETFYQVAFLSSAMPSSTGRDGLFHILAQNQRILDFCSKAGLGAKQYMPHYS
PpCKX1 NTSLVTPDEDVFYLVAFLSSAVPSSTGTDGLDQILTQNTRILEYCDRAQLGIKQYLPYYR
AtCKX2 RMSAMIPDEDVIYIIGLLQ-----SAT-KDLPEVESVNEKIIRFCKDSGIKIKQYLM---
AtCKX3 RMSAAIPEEDVFYAVGFLR-----SAG-DNWEAFDQENMEILKFCEDANMGVIQYLPYHS
PtCKX3a KMSAVIPEEDIFYTVGFLH-----SSG-NDWQAYDHQNKDILKFCDKAGIEIKQYLPLYN
PtCKX3b ELSAVIPDEDIFYTTSFLH-----TSG-DNWQVYEDQNQAVIKFCEEAG-----------
AtCKX4 RMSTMTPDEDVFYVIGLLQ-----SAGGQNWQELENLNDKVIQFCENSGIKIKEYLMHYT
AtCKX5 RSSAVTPDEEVFYLVALLRSALTDGEETQKLEYLKDQNRRILEFCEQAKINVKQYLPHHA
PtCKX5a RSSVVTPDEDVFYLVALLRSALDNGEETQSLEYLTNQNHKILRFCDDAGIKVKQYLPHYT
PtCKX5b RSSVVTPDEDVFYLVALLRSALDNGEETQSLEYLTDQNRKILRFCDDAGIKVKQYLPHYT
PpCKX5 RSSVVTPDEDIFYLVALLRSALDTGDETHTLEHLTNQNRQILKFCDDAGIKVKQYLPHYT
AtCKX6 QTSAVTPEEEVFYLVAILTSASPGSAGKDGVEEILRRNRRILEFSEEAGIGLKQY-----
PtCKX6 RTSAVIPEENIFYLVAFLTSAVPSSTGTDGLEHILTQNKRILEFCEIARLGMKQYLPHYT
PpCKX6 RTSVVIPEEHIFYLVAFLTSAVPSSTGTDGLEHILTQNKRILEFCETAHLGVKQYLPHYT
AtCKX7 RTSVVIPEGEIFYIVALLR-----FVPPCSVEKMVAQNQEIVHWCVKNGIDYKLYLPHYK
PtCKX7 RTSVVLPEGEIFYLVALLR-----FTMPCSAEKLVSQNREIVQFCVKEGLDFKLYLPHYQ
PpCKX7 RTSVVLPESEIFYIVALLRFT-PPYPKGPSFKNLVAQNKEIIQYCNKKGFDFKLYLPHYR
PpCKX3 RMSAVVPEEEVFYTVGFLH-----SSGFDEWEALEEQNKEILKFCASVGIEIKQYLPYYK
PpCKX2 RMSAVIPDEDVFYVVGLLH-----SSG---------FNTQLLQFCHDAGIMVKQYLPHYQ
AtCKX1 TQKEWKSHFGK
PtCKX1a TQEEWQTHFGP
PtCKX1b TQEEWQAHFGP
PpCKX1 TQQEWKAHFGP
AtCKX2 -----------
AtCKX3 SQEGWVRH---
PtCKX3a SNKEWINHFG-
PtCKX3b -----------
AtCKX4 RKEDWVKHFGP
AtCKX5 TQEEWVAHFGD
PtCKX5a TQEEWMDHFGD
PtCKX5b TREEWMDHFGD
PpCKX5 TQEEWMDHFGD
AtCKX6 -----------
PtCKX6 THEEWKAHFGP
PpCKX6 TKKEWQAHFGP
AtCKX7 SQEEWIRHFGN
PtCKX7 SEEEWKRHFGS
PpCKX7 SQDDWKRHFGN
PpCKX3 TQQGWMNHFGS
PpCKX2 TQEDWKNHFGS
