# Supplementary material for: Characterization of cytokinin signaling and homeostasis gene families in two hardwood tree species: Populus trichocarpa and Prunus persica
Source: BMC Genomics. 2013 Dec 16;14:885. doi: 10.1186/1471-2164-14-885 (PMC3866579; doi:10.1186/1471-2164-14-885)
Supplement: Additional file 5: Figure S2 — Alignment of Populus (Pt), Prunus (Pp) and Arabidopsis isopentenyl transferases (IPTs). [file 1471-2164-14-885-S5.doc]

**Supplementary Figure 2** Alignment of *Populus* (Pt), *Prunus* (Pp) and Arabidopsis isopentenyl transferases (IPTs).

PpIPT2 22 KPKVVVIMGPTGSGKSKLAIDLASYFPIEVINADSMQVYNGLDVLTNKVPLHEQKGVPHH
PtIPT2 34 KPKLVVIMGPTGSGKSKLAIDLAAHFPVEIINADSMQVYRGLDVLTNKVPISDQEGVPHH
AtIPT2 19 KAKVVVIMGPTGSGKSKLAVDLASHFPVEIINADAMQIYSGLDVLTNKVTVDEQKGVPHH
AtIPT1 64 KDKVVVILGATGAGKSRLSVDLATRFPSEIINSDKIQVYEGLEITTNQITLQDRRGVPHH
AtIPT4 04 NDKMVVIMGATGSGKSSLSVDLALHFKAEIINSDKMQFYDGLKITTNQSTIEDRRGVPHH
AtIPT8 42 KQKVVVIMGATGSGKSCLSIDLATRFSGEIVNSDKIQFYDGLKVTTNQMSILERCGVPHH
AtIPT6 44 KDKVVLITGTTGTGKSRLSVDLATRFPAEIINSDKMQIYKGFEIVTNLIPLHEQGGVPHH
PtIPT6b 18 KDKILVIMGATGCGKTKVSIDLATRFHSEIINSDKIQVYKGLDIITNKIPVQDRLGVPHY
PtIPT6a 60 KDKILVIMGATGCGKTRVSIDLATRFQSEIINSDKMQVYEGLDITTNKITIQDRLGVPHH
PpIPT6 18 KDKLLIIMGATGAGKSRLSLDLATRFPSEIVNSDKMQLYAGLDITTNKLPIPDRLGVPHH
PtIPT3 35 KEKVVIVMGPTGTGKSRLSIELATQFPAEIINSDKMQVYKGLDIVTNKVAEEEKSGVPHH
PpIPT3 30 KEKVVIVMGATGTGKSRLSIDLATHLAAEIINSDKMQVYKGLDIATNKITEEEQRGVPHH
AtIPT3 40 KDKVVVIMGATGTGKSRLSVDIATRFRAEIINSDKIQVHQGLDIVTNKITSEESCGVPHH
PtIPT5a 32 KDKVVFVLGPTGTGKSRLAIDLATHFPAEVVNCDKMQVYKGLDIVTNKVTEEECRGVPHH
PtIPT5b 32 KDKVVFVVGPTGTGKSRLAIDLATRFPAEVVNCDKMQVYKGLDIVTNKVTEEECRGVPHH
AtIPT5 32 KDKVVFVMGATGTGKSRLAIDLATRFPAEIVNSDKIQVYKGLDIVTNKVTPEESLGVPHH
PpIPT5a 35 KDKVVFVMGATGTGKSRLAIDLATFHRAEIINSDKMQVYKGLDIVTNKVTEEECRGIPHH
PpIPT5b 08 KDKVVFVMGATGTGKSRLAIDLATFHRAEIINSDKMQVYKGLDIVTNKVTEEECRGIPHH
AtIPT7 33 KEKVIFVMGATGSGKSRLAIDLATRFQGEIINSDKIQLYKGLDVLTNKVTPKECRGVPHH
PtIPT7b 52 KQKALFVMGTTATGKSKLSIDLATHFQGEIINSDKIQVYKGLDILTNKVSEDESRGVPHH
PtIPT7a 52 KQKALFVMGTTATGKSKLSIDLATHFQGEIINSDKIQVYKGLDMLTNKISEIERRGVPHH
PpIPT7 24 KTKVIFVLGSTGCGKTKLSIDLATRYNGEIINSDKIQVYKGLDIVTNKATKPEQRGILHH
PtIPT9 46 KEKLIVISGPTGSGKTRLALELAKRLNGEIVSADSVQVYRGLDVGSAKPTESERKEVRHH
PpIPT9 53 KEKVIVVSGPTGAGKSRLAFELAKRLNGEIISADSVQVYRGLDIGSAKPSPGDRQEVPHH
AtIPT9 48 KEKVIVISGPTGAGKSRLAMELAKRLNGEIISADSVQVYKGLDVGSAKPSDSDRKVVPHH

PpIPT2 LLGTVSPNV-EFTAKDFRDFSIPLIDDILSRNCLPVIVGGTNYYIQALVSPFLLDDTVED
PtIPT2 LLGTLNPNV-EFTAKDFRDSAIPLINEILSRNCLPVVVGGTNYYIQALVSPFLLDD----
AtIPT2 LLGTVSSDM-EFTARDFRDFTVPLIEEIVSRNHIPVLVGGTHYYIQAVVSKFLLDD----
AtIPT1 LLGVINPEHGELTAGEFRSAASNVVKEITSRQKVPIIAGGSNSFVHA------LLAQRFD
AtIPT4 LLGELNPEAGEVTAAEFRVMAAEAISEITQRKKLPILAGGSNSYIHA------LLAKSYD
AtIPT8 LLGELPPDDSELTTSEFRSLASRSISEITARGNLPIIAGGSNSFIHA------LLVDRFD
AtIPT6 LLGQFHPQDGELTPAEFRSLATLSISKLISSKKLPIVVGGSNSFNHA------LLAERFD
PtIPT6b LLGEFDPEDGELTLSEFRLAGGLAISGIVSRQRLPIVVGGSNSLVHA------LVVDRFN
PtIPT6a LLGEFDPDDGELTPSEYRLAGGLAISGIVSRQNLPIVVGGSNSLIHA------LVVDRFN
PpIPT6 LLGEFDPRHGDFTPSQFRAVAGQAISSITNRRKVPMLVGGSNSFIHA------LLVDRFE
PtIPT3 LLGIANPTV-DFTATNYCHTASLAVESISTRGLLPIIVGGSNSYIEA------LMDDEDF
PpIPT3 LLGILDPNE-DFTATDFCDETSLTIESILGRDRLPIIVGGSNSYIEA------LIDD---
AtIPT3 LLGVLPPEA-DLTAANYCHMANLSIESVLNRGKLPIIVGGSNSYVEA------LVDDKEN
PtIPT5a LLGIADPNA-DFTSDDFRHHASLVVESIVTRDRLPIIAGGSNSYVEA------LANDDPE
PtIPT5b LLGIADPNA-NFTSDDFRHHASLVVESIVTRDRLPIIAGGSNSYIEA------LANDDPE
AtIPT5 LLGVHDTYE-DFTAEDFQREAIRAVESIVQRDRVPIIAGGSNSYIEA------LVNDCVD
PpIPT5a LLGQIDPNF-NFTANDFKQHASLAIESILERDRLPIIAGGSNSYIEA------LVDDHPE
PpIPT5b LLGQIDPNF-NFTANDFKQHASLAIESILERDRLPIIAGGSNSYIEA------LVDDHPE
AtIPT7 LLGVFDSEAGNLTATQYSRLASQAISKLSANNKLPIVAGGSNSYIEA------LVNHSSG
PtIPT7b LLGFVE-PGEEFTTQDFCNHVHMAMRHIIGNGNIPIIAGGSNRYIEA------LVEDPLF
PtIPT7a LLGFVE-PGEEFTTQDFCNHVHKAMKHITGDGSIPIIAGGSNRYIEA------LVEDPLF
PpIPT7 LLGSIQDPEADFTVQDFCLQVPKALDEITKRNRVPIIAGGSNTYIEA------LVED---
PtIPT9 LLDILHPSE-DYSVGQFYEDARQATQDILKNGRVPIVTGGTGLYLR--------------
PpIPT9 LVDILHPSE-DYSVGKFYEDARQATRSILDSGRVPVVTGGTGLYLR--------------
AtIPT9 LIDILHPSQ-DYSVGQFYDDGRQATKDILNRGRVPIVTGGTGLYLR--------------

PpIPT2 MDESVDNFRYNCCFICVDASLPVLDQFVEQRVDCMIDAGLLNEVYEIFTRN---ADYTRG
PtIPT2 -----TNYRFHCCFICVDADIPVLDRYVEQRVDSMIDAGLLGEVCEVYNYN---ADYTRG
AtIPT2 -----AASRFDYCLICMDAETAVLDRYVEQRVDAMVDAGLLDEVYDIYKPG---ADYTRG
AtIPT1 PKFDPFSDLRYECCFIWVDVSETVLYEYLLRRVDEMMDSGMFEELSRFYDPVK-ETR-FG
AtIPT4 PENYPFSELKYDCCFIWIDVDQSVLFEYLSLRLDLMMKSGMFEEIAEFHRSKK-PKEPLG
AtIPT8 PKTYPFSGLRYECCFLWVDVSVSVLFEYLSKRVDQMMESGMFEELAGFYDPRY-AIRAHG
AtIPT6 PDIDPFSDLRYKCCILWVDVLEPVLFQHLCNRVDQMIESGLVEQLAELYDPVV-SGRRLG
PtIPT6b PELNVSTQLRYNCCFLWVDVSLPVLCDYLCMRVDEMLDSGMFDELSEYYGSID-SASQTV
PtIPT6a PELNVFTQLRYNCCFLWVDVSLPVLCDYLCKRVDEMLDSGMLDELSEYYGSVD-AASQIG
PpIPT6 PGSNVFEELRYNCCFLWVDVSLAVLTEYLCKRVDEMLDSGMLDELAEFCDPDT------A
PtIPT3 ----RLRLN-YDCCFLWVDVSMPVLHKFVSRRVEQMVSVGMIDEVRNIFDP---ADYSTG
PpIPT3 -----FRSK-YECCFLWVNVSTPVLHSFVSKRVDKMVENGMVDEVREFFHPN--ADYSKG
AtIPT3 ----KFRSR-YDCCFLWVDVALPVLHGFVSERVDKMVESGMVEEVREFFDFS--SDYSRG
PtIPT5a -----FRLR-YECCFLWVDVSLPLLHSFVSDRVDRMVRAGLIDEVRDVFDP-TKDDYSQG
PtIPT5b -----FRLR-YECCFLWVDVSLPILYSFVSERVDRMVEAGLIDEVRDMFDP-NKDDYSQG
AtIPT5 -----FRLR-YNCCFLWVDVSRPVLHSFVSERVDKMVDMGLVDEVRRIFDP-SS-DYSAG
PpIPT5a -----FRMK-YECCFIWVDVALPVLNSFVSERVDRMVKAGLVDEVRRMFDSTAE-EYTHG
PpIPT5b -----FRMK-YECCFIWVDVALPVLNSFVSERVDRMVKAGLVDEVRRMFDSTAEAEYTHG
AtIPT7 -----FLLNNYDCCFIWVDVSLPVLNSFVSKRVDRMMEAGLLEEVREVFNPKA--NYSVG
PtIPT7b -------KDNYDTCFLWVDVALPILFVRAAKRVDKMLDAGLVDEVRGMFIPG--IDHNSG
PtIPT7a -----NFKDSYDTCFLWVDVALPILFDRAAKRVDEMLDAGLVEEVRGMFIPG--IDHNSG
PpIPT7 -------PDKYDCCFIWLDVSLPVLYNRVSERVDEMVDAGLVDELREMFVPG--ADYERG
PtIPT9 -------------WFIYGKPDVPKASPYRLRRSLEIIKTS--GSPPSAFDSSL---PRLD
PpIPT9 -------------WLIYGKPDVPKASPYRLRRSLEIIKSS--GSPPSAFDSSL---KRVD
AtIPT9 -------------WFMYGKPDVPKPSPYRLRRSLEILKST--GSPPSSFDDFL---PRVA

PpIPT2 LRQAIGVREFEIFLRAYITDISDSQPKIALNEAIDKMKMNTRRLVRRQKRRINRLEALFG
PtIPT2 LRQAIGVREFDNFLRSSD----DNQLKILLAEAIDKVKANTRRLVRVQKRRLTRLQTFFG
AtIPT2 LRQSIGVREFEDFLRFPK----DDKLRIMLEEAIDRVKLNTRRLLRRQKRRVSRLETVFG
AtIPT1 IRKAIGVPEFDGYFMIKWD----ALRKAAYDKAVDDIKRNTWTLAKRQVKKIEMLKDAG-
AtIPT4 IWKAIGVQEFDDYLMDKWD----PMRKEAYEKAVRAIKENTFQLTKDQITKINKLRNAG-
AtIPT8 IHKTIGIPEFDRYFMSEWD----QARKGAYDEAVQEIKENTWRLAKKQIERIMKLKSSG-
AtIPT6 VRKTIGVEEFDRYFKGIWD----LARKAAYEETVKGMKERTCRLVKKQKEKIMKLIRGG-
PtIPT6b LRKAIGVPEFDRYFKKYPPGEWDQVRRGIYEECVREIKENTCQLAKRQIGKILRLKGAG-
PtIPT6a LRKAIGVP----EFDRYF-GEWD-RRRGVYEVCVREIKENTCQLAKRQIGKILRLKGAG-
PpIPT6 LRKAIGVPEFTRYFKRD--------RRGAYEEAVRAIKDNTCQLAKRQIGKILRLKGGG-
PtIPT3 IRRSIGVPEFDKYFRAEAF-LDEENRARLLHEAICDVKKNTCKLACRQWEKINRLRKIKG
PpIPT3 IRRAIGVPEFDKYFRF----LDEETKARLLEQAVEEIKKNNCKLASRQLEKIQRLRNVKG
AtIPT3 IKKAIGFPEFDRFFRNEQF-LNVEDREELLSKVLEEIKRNTFELACRQREKIERLRKVKK
PtIPT5a IKRAIGVPELDQFLRNETI-VDAKTRRKLLDEAIEKIKENTCMLARRQLQKIRRLHSIWN
PtIPT5b IKRAIGVPELDHFLRNEAI-VDAKTRRKLLDEAIDKIKENTCMLASRQLQKIHRLHSIWN
AtIPT5 IRRAIGVPELDEFLRSEMRNYPAETTERLLETAIEKIKENTCLLACRQLQKIQRLYKQWK
PpIPT5a IRKAIGMPELDEYLRSEACGNDEETQERLLQTAISKIKENTCILAHRQLKKIRRLCSRRN
PpIPT5b IRKAIGMPELDEYLRSEACGNDEETQERLLQTAISKIKENTCILAHRQLKKIRRLCSRRN
AtIPT7 IRRAIGVPELHEYLRNESL-VDRATKSKMLDVAVKNIKKNTEILACRQLKKIQRLHKKWK
PtIPT7b IWRAIGIPELEPYFQAEMEMADEVTRKMLLDTGIKEMKENTKKLINKQLRKIKYLANEKG
PtIPT7a IWRAIGIAEMEPYFQAEMEMADEVTMKILLETGIKEMKENTKKLINKQLTKIKYLANKKG
PpIPT7 IRRAIGAPEMHAYFMAEMD--DEAGKEFLFKDGIQKTKDNTLKLAESQVQKIERLGTK--
PtIPT9 LYKSIDYRCEDMVAWLLDE--GLLPNSNSATRAIGYRQAMEYLLRCREDGGSSSAGDFYA
PpIPT9 LYRSIDCRCEDMLS-------GLLPNSNSATRAIGYRQAMEYILMCRQQGGSSPR-EFFN
AtIPT9 LYRSIDFRCEDMLSWLLDL--GLLPNSNPATRAIGYRQAMEYLLQCRRYEGESSPREFYA

PpIPT2 WNIHFLDATKFIL---CKSDDTWAAEVVGPAVEMIRSFLNRD
PtIPT2 WNIHYVDATEFIS---CKTDELWAGQVVSSAVNVIRAFLTEE
AtIPT2 WNIHYIDATEYIL---SKSEESWNAQVVKPASEIIRCFLETE
AtIPT1 WEIERVDATASF--AVKKWRENWEEQVLEPSVKIVKRHLVQN
AtIPT4 WDIKKVDATASFR-AVEMQRKIWNKEVLEPCVKIVRSHLDQP
AtIPT8 WDIQRLDATPSF------GREIWDNTVLDESIKVVKRFLVKD
AtIPT6 WEIKRLDATAAIM-EEKNGREIWEKHIVDESVEIVKKFLLEV
PtIPT6b WDLQRVDATESF---REVWVEVWRRDVMEPSMKIVKRFLDE-
PtIPT6a WDLKRVDATESF---REVWMEVWGRDVMEPSMKIVKRFLEEE
PpIPT6 WDLQRLDATDAFRADGKRWSEIWERQVVKPSVKVVKRFLEE-
PtIPT3 WDIHRLDATEVFQ-KSKEADHAWEMLVARPSTAIVGQLLCGV
PpIPT3 WNLHPLDATEVFR-KRKESDEAWEKFVYGPSAQIVRQFLYNY
AtIPT3 WSIQRVDATPVFT-KRMDANVAWERLVAGPSTDTVSRFLLDI
PtIPT5a WKMHRIDATPVFL-ASKEADNLWDQIVAGPSTMIVNQFLC--
PtIPT5b WNVHRIDATPVFL-TSKEVDNLWDKLVAGPSTMIVNQFLC--
AtIPT5 WNMHRVDATEVFL-RREEADEAWDNSVAHPSALAVEKFLSYS
PpIPT5a WNMHRLDATEVFL-HRVEADQAWEKHVARPAKRMVGNFLVDP
PpIPT5b WNMHRLDATEVFLKHRVEADQAWEKHVARPAKRMVGNFLVDP
AtIPT7 MSMHRVDATEVFL-KREEQDEAWENLVARPSERIVDKFYN--
PtIPT7b WKLHRIDATFVYERSGNVDEDVWDDKVLRPSLEMLTNFLQED
PtIPT7a WKFHRIDATCVYE-RSKVDEDVWDKKVLRPSLEIVTNFLRED
PpIPT7 WDIHRIDVTAVHE-RGKKAVVAWENLVLKPSFSIVSEFLEMD
PtIPT9 FLSEFQKASRNFA-KRFRNEHIYHWLDASKPLEMVLNFVYDA
PpIPT9 FLSEFQKASRNFA-KRFRNESIYHWLDASKPLETVLNFIYDA
AtIPT9 FLNKFQTASRNFA-KRFRCEPMYHWLNASKPLDSILQCIYDA
