# Supplementary material for: Characterization of cytokinin signaling and homeostasis gene families in two hardwood tree species: Populus trichocarpa and Prunus persica
Source: BMC Genomics. 2013 Dec 16;14:885. doi: 10.1186/1471-2164-14-885 (PMC3866579; doi:10.1186/1471-2164-14-885)
Supplement: Additional file 6: Figure S3 — Alignment of Populus (Pt), Prunus (Pp) and Arabidopsis LONELY GUY (LOG) proteins). [file 1471-2164-14-885-S6.doc]

**Supplementary Figure 3** Alignment of *Populus* (Pt), *Prunus* (Pp) and Arabidopsis LONELY GUY (LOG) proteins.

AtLOG1 05 SKFKRICVFCGSSAGNKVSYKDAAIELGTELVSRNIDLVYGGGSIGLMGLISQAVFNGGR
PtLOG1 08 SRFKRICVFCGSSPGKKSSYKDAAIELGKELVSRNIDLVYGGGSIGLMGLISQAVFDGGR
PpLOG1 08 RSPIKVCVFCASSPRKKSSYKEAAIELGEELVSKNIDLVYGGGSVGLMGLISQAVYDGGR
AtLOG2 06 SRFRRICVFCGSSSGNKTTYHDAALQLAHQLVERNIDLVYGGGSVGLMGLISQAVHDGGR
AtLOG3 11 SKFRRICVFCGSSQGKKSSYQDAAVDLGNELVSRNIDLVYGGGSIGLMGLVSQAVHDGGR
AtLOG4 11 SKFGRICVFCGSSQGKKSSYQDAAVDLGNELVLRNIDLVYGGGSIGLMGLVSQAVHDGGR
AtLOG5 06 SRFKRVCVFCGSSSGKRECYSDAATDLAQELVTRRLNLVYGGGSIGLMGLVSQAVHEAGG
PtLOG5a 11 SKFKRVCVFCGSSTGKRDCYRDAALELGQELVSRSLDLVYGGGSVGLMGLVSQEVHRGGG
PtLOG5b 09 SRFKSVCVFCGSSTGKRNCYRDAAIELAQELVAKRLDLVYGGGSIGLMGLVSQAVHRGGG
PtLOG5c 11 SRFKRVCVFCGSSKGKRDCYRDAALELGQELVSRRLDLVYGGGSVGLMGLVSQEVHRGGG
PtLOG5d 09 SRFKRVCVFCGSSTGKRKCYRDAATELGQELVAKRLDLVYGGGSIGLMGLVSQAVHSGGG
PpLOG5 08 SRFKRVCVFCGSSTGKRNCYKDAAIELAQELVSRRLDLVYGGGSIGLMGLVSQAVHRGGG
AtLOG6 16 SRFKSICVFCGSSNGNKASYQDAAIDLAKELVMRKIDLVYGGGSIGLMGLVSQAVHDGGR
PtLOG6 18 SKFKRICVFCGSSQGKKTSYQVAAIDLGNELVSRNIDLVYGGGSIGLMGLVSQAVHDGGR
PpLOG6 08 SRFGRICVFCGSSQGKKSSYQDAAIELGKELVSRNIDLVYGGGSIGLMGLVSQAVHDGGR
AtLOG7 06 SRFKRICVFCGSSSGKKPSYQEAAIQLGNELVERRIDLVYGGGSVGLMGLVSQAVHHGGR
PtLOG7a 13 SRFRRVCVFCGSSPGKNPNYQHAAIQLGKQLVERNIDLVYGGGSIGLMGLVSQAVYDGGR
PtLOG7b 06 SKFKRVCVFCGSSSGKKASYQEAAVELAKELVERRIDLVYGGGSVGLMGLVSQAVHDGGR
PpLOG7a 08 SRFNRICVFCGSSSGKKASYQEAAVELGKELVERRIDLVYGGGSVGLMGLVSQAVHDGGR
PpLOG7b 08 SRFKRLCVFCGSSPGKNPSYQLAAIQLGKQLVERNIDLVYGGGSIGLMGQVSQAVYDGGR
AtLOG8 07 SRFRKICVFCGSHSGHREVFSDAAIELGNELVKRKIDLVYGGGSVGLMGLISRRVYEGGL
PtLOG8a 15 RRLRRICVFCGSRAGYKSSFSDAALELGKQLVKRKIDLVYGGGSGGLMGLISQTVFNGGC
PtLOG8b 02 GKFTRVCVFCGSKSGNKKIFSDAALDLGRQLVERKMDLVYGGGSVGLMGLVSQTVYDGGS
PtLOG8c 02 GKFKSVCVFCGSKSGNKKIFSDAALDLGRELVERKMDLVYGGGSIGLMGLVSQTVYDGEC
PtLOG8d 09 SKFKRVCVFCGSNSGNRQVFSDAAIELGDELVKRKIELVYGGGSVGLMGLISQKVYDGGC
PtLOG8e 15 GRFKRICVFCGSRAGYKSSFSDASLELGKQLVRRKIDLVYGGGSAGLMGLISRTVFNGGC
PpLOG8a 08 SKFKRICVFCGSNSGRRKVFSDAALELGNELVKRKIDLVYGGGSVGLMGLISQTVYDGGC
PpLOG8b 08 GKFKRVCVFCGSNSGHRKIFSDAALQLGTQLVERKMDLVYGGGSVGLMGLVSQTVYDGGC
AtLOG9 04 -----------------------------------------------------------M

AtLOG1 HVIGVIPKTLMPREITGETVGEVKAVADMHQRKAEMAKHSDAFIALPG-GYGTLEELLEV
PtLOG1 HVIGVIPKTLMPREITGETVGEVKAVADMHQRKAEMARHSDAFIALPG-GYGTLEELLEV
PpLOG1 HVIGVIPKTLMPREITGETVGEVKAVADMHQRKAEMAKHSDAFIALPG-GYGTLEELLEV
AtLOG2 HVLGIIPKSLAPREITGESIGEVITVSTMHQRKAEMGRQADAFIALPG-GYGTFEELLEV
AtLOG3 HVIGIIPKTLMPRELTGETVGEVRAVADMHQRKAEMAKHSDAFIALPESGYGTLEELLEV
AtLOG4 HVIGVIPKTLMPRELTGETVGEVRAVADMHQRKAEMARHSDAFIALPG-GYGTLEELLEV
AtLOG5 HVLGIIPRTLMDKEITGETYGEVIAVADMHERKAEMARHSDCFIALPG-GYGTLEELLEV
PtLOG5a HVIGVIPKTLMSKELTGETVGEVRPVADMHQRKAEMARHSDCFIALPG-GYGTLEELLEV
PtLOG5b NVLGIIPRTLMSKEITGETVGEVKPVADMHQRKAEMARNSDCFIALPG-GYGTLEELLEV
PtLOG5c HVIGVIPKTLMNKELTGETVGEVRPVADMHQRKAEMARNSDCFIALPG-GYGTLEELLEV
PtLOG5d NVLGIIPRTLMSKEITGETVGEVKPVADMHQRKAEMARNSDCFIALPG-GYGTLEELLEV
PpLOG5 NVLGIIPRTLMCKEITGETVGEVRPVADMHQRKAEMARHSDCFIALPG-GYGTLEELLEV
AtLOG6 HNNNSVNVSQTNSKLTGETVGEVKEVADMHQRKAVMAKHSDAFITLPG-GYGTLEELLEV
PtLOG6 HVIGVIPKTLMPRELTGETVGEVKAVADMHQRKAEMAKHSDAFIALPG-GYGTLEELLEV
PpLOG6 HVIGVIPKTLMPRELTGETVGEVKAVAGMHQRKAEMAKHSDAFIALPG-GYGTLEELLEV
AtLOG7 HVLGVIPKTLMPREITGETIGEVKAVADMHQRKAEMARQADAFIALPG-GYGTLEELLEV
PtLOG7a HVLGVIPKTLMPREITGDTVGEVKAVSGMHQRKAEMARQADAFIALPG-GYGTLEELLEV
PtLOG7b HVLGVIPRSLMPREVTGEPVGEVRAVSDMHQRKAEMARQADAFIALPG-GYGTLEELLEV
PpLOG7a HVLGVIPRTLMPRELTGETVGEVRTVSDMHQRKAEMARQADAFIALPG-GYGTLEELLEV
PpLOG7b HVLGVIPRTLMPREITGEPVGEVRAVSGMHQRKAEMARQADAFIALPG-GYGTLEELLEV
AtLOG8 HVLGIIPKALMPIEISGETVGDVRVVADMHERKAAMAQEAEAFIALPG-GYGTMEELLEM
PtLOG8a HVLGVIPKALMSHEISGETVGEVIAVADMHQRKAEMAKHADAFIALPG-GYGTMEELLEI
PtLOG8b HVLGIIPTALVPIEISGETVGEVLIVSDMHERKAEMARRADAFIALPG-GYGTFEELLEM
PtLOG8c HVLGVIPRALVPIEISGHTVGEVLIVSDMHERKAEMARRADAFIALPG-GYGTFEELLEM
PtLOG8d HVLGVIPKALMPLEISGQTVGEVRTVVDMHERKAVMAKESDAFIALPG-GYGTMEELLEM
PtLOG8e HVLGVIPKALMSHEISGEAVGEVKTVADMHQRKAEMAKHADAFIALPG-GYGTMEELLEI
PpLOG8a HVLGVIPKALMPLEISGPTVGEVRTVTDMHERKASMAQEADAFIALPG-GYGTMEELLEI
PpLOG8b HVLGVIPTALVPLEISGNSIGEVLIVSDMHERKAEMARQADAFIALPG-GYGTMEELLEM
AtLOG9 HIE----------HISGETVGEVRIVSDMHERKATMAQEAGAFIALLGERYETMEELLEM

AtLOG1 ITWAQLGIHDKPVGLLNVEGYYNSLLSFIDKAVEEGFISPTARHIIVSAPSAKELVKKLE
PtLOG1 ITWAQLGIHDKPVGLLNVDGYYNSLLSFIDKAVEEGFINPSARHIIVSAPTPRELVKKME
PpLOG1 ITWAQLGIHDKPVGLLNVDGFYNSLLSFIDKAVEEGFISPTARHIIVSAPTAKELVKKME
AtLOG2 ITWSQLGIHTKPVGLLNVDGFYDSLLTFIDKAVDEGFVSSTARRIIVSAPNAPQLLQLLE
AtLOG3 ITWAQLGIHDKPVGLLNVDGYYNSLLSFIDKAVEEGFISPTAREIIVSAPTAKELVKKLE
AtLOG4 ITWAQLGIHDKPVGLLNVDGYYNSLLSFIDKAVEEGFISTNARQIIISAPTAKELVKKLE
AtLOG5 IAWAQLGIHDKPVGLLNVDGYYNYLLTFIDKAVDDGFIKPSQRHIFVSAPNAKELVQKLE
PtLOG5a ITWAQLGIHDKPVGLLNVDGYYNYLLTFIDKAVDDGFIMPSQRSIIVSAPNAKELVQKLE
PtLOG5b ITWAQLGIHDKPVGLLNVDGYYNYLLTFIDKAVDDGFIKPSQRNIIVSAPSAKELVQKLE
PtLOG5c ITWAQLGIHDKPVGLLNVDGYYNYLLTFIDKAVDDGFIMPSQRSIIVSAPSPKELVQKLE
PtLOG5d TTWAQLGIHDKPVGLLNVDGYYNYLLTFIDKAVDDGFIKPSQRNIIVSAPNARELVQKLE
PpLOG5 ITWAQLGIHDKPVGLLNVDGYYNYLLTFIDKAVDDGFIKPSQRHIIVSAPNAKELVQKLE
AtLOG6 ITWAQLGIHDKPVGLLNVDGYYDALLLFIDKAVEEGFILPTARHIIVSAPTARELFIKLE
PtLOG6 ITWAQLGIHDKPVGLLNVDGYYNSLLSFIDKAVEEGFINPSARNIIVSAPTAKELVKKLE
PpLOG6 ITWAQLGIHDKPVGLLNVDGYYNSLLSFIDKAVEEGFISPSARHIIISAPTARELVKKLE
AtLOG7 ITWAQLGIHRKPVGLLNVDGYYNSLLTFIDKAVDEGFISPMARRIIVSAPNAKELVRQLE
PtLOG7a ITWAQLGIHDKPVGLLNVDGYYNSLLSFIDKAVDEGFITPAARHIIVSANTAQELMCLLE
PtLOG7b ITWAQLNIHHKPVGLLNVDGYYNSLLSFIDKAVDEGFISPAARRIIVSASTAKQLFRQLE
PpLOG7a ITWAQLGIHRKPVGLLNVDGFYNSLLSFIDKAVDEGFISTTARQIIVSAPTAKQLVRQLE
PpLOG7b ITWAQLGIHDKPVGLLNVDGYYNSLLSFIDKAVDEGFIDPAARHIIVSAQTAQELMCKLE
AtLOG8 ITWSQLGIHKKTVGLLNVDGYYNNLLALFDTGVEEGFIKPGARNIVVSAPTAKELMEKME
PtLOG8a IAWSQLGIHEKPVGLLNADGYYDSLLALFDKGVEEGFIRDTARHIVITAETAAELIEKME
PtLOG8b ITWSQLGIHNKPVGLLNVDGYYDSLLGLFDKSVEEGFVNASARNIVVSARTARELIQRME
PtLOG8c ITWSQLGIHNKPVGLLNVDGYYDSLLGFFDKGVEEGFIGPSARNIVISARTATELIQKME
PtLOG8d ITWSQLGIHKKPVGLLNVDGYYNCLLALFDNGVEQGFIKPGARDIVVSAPTAKELMEKME
PtLOG8e ISWSQLGIHEKPVGLLNVDGYYNSLLALFDKGVEEGFINDTARHIVVIAETAAELIKKME
PpLOG8a ITWAQLGIHMKPVGLLNVDGYYNSLLALFDNGVEEGFIKPCARHIVVSAPTAKELVVKME
PpLOG8b ITWSQLGIHDKPVGLLNIDGYYDCLLRLFDKGVEEGFINLSARNIVISAKTAQELIQRME
AtLOG9 ITWAQLGIHKKTVGLLNVDGYYNNLLAFFDTGVEEGFIKQGACNIVVSAPSARELMEKME

AtLOG1 DYVPRHEKVASKKSWEME
PtLOG1 EYFPRHEIVASKLSWEIE
PpLOG1 EYFPRHERVASKLSWEIE
AtLOG2 EYVPKHDDFVSKMVWDNT
AtLOG3 EYAPCHERVATKLCWEME
AtLOG4 EYSPCHESVATKLCWEIE
AtLOG5 AYKPVNDGVIAKSRWEVE
PtLOG5a EYVPVHDGVVAKAKWEAE
PtLOG5b EYVPVHDGVIAKASWEIE
PtLOG5c EYVPVHDGVVAKAKWEAE
PtLOG5d EYVPVLDGVIAKASWEIE
PpLOG5 EYVPVHDGAIAKARWEVE
AtLOG6 LNMVSLDRISKHALSLFQ
PtLOG6 EYVPCHERVASKLSWEIE
PpLOG6 DYVPCHERVASKLNWEME
AtLOG7 EYEPEFDEITSKLVWDEV
PtLOG7a DYEAEHSGVASKLSWEMG
PtLOG7b DYVPEHDEITAKLVWGEV
PpLOG7a EYVPEQDEITSKLVWEEV
PpLOG7b EYIPKHSG----LSWEME
AtLOG8 EYTPSHMHVASHESWKVE
PtLOG8a QYAPVHDKVAPRQSWEVD
PtLOG8b DYIPVHEQVTSNQSCNVE
PtLOG8c DYIPLHEQVAPSHSWKVE
PtLOG8d LYTPSHKQVAPRESWNME
PtLOG8e EYAPVHDKVAPRQSWEVD
PpLOG8a QYTPSHEHVASHESWQME
PpLOG8b EYIPLHNQVAPTQSWNVE
AtLOG9 LYTPSHKYIASHQSWKVE
