# Supplementary material for: Characterization of cytokinin signaling and homeostasis gene families in two hardwood tree species: Populus trichocarpa and Prunus persica
Source: BMC Genomics. 2013 Dec 16;14:885. doi: 10.1186/1471-2164-14-885 (PMC3866579; doi:10.1186/1471-2164-14-885)
Supplement: Additional file 7: Figure S4 — Alignment of Populus (Pt), Prunus (Pp) and Arabidopsis CRE1- and CKI1-like two-component histidine kinase, together with Arabidopsis ethylene receptors (ETR1, ETR2, ERS1, ERS2 and EIN4), phytochromes (PHYA-E), a putative osmosensor (AtHK1), and the histidine kinase CKI2/AHK5. [file 1471-2164-14-885-S7.doc]

**Supplementary Figure 4** Alignment of *Populus* (Pt), *Prunus* (Pp) and Arabidopsis CRE1- and CKI1-like two-component histidine kinase, together with Arabidopsis ethylene receptors (ETR1, ETR2, ERS1, ERS2 and EIN4), phytochromes (PHYA-E), a putative osmosensor (AtHK1), and the histidine kinase CKI2/AHK5.

AHK1 ANMSHELRTPMAAVIGLGDSARLVQIFANLISNSIKFEVDDTGCGIDVFESFEQGTGLGL
AHK2 ATVSHEIRTPMNGVLGMGDPSRFRQIITNLVGNSIKVTVEDTGVGIPIFTPFMQGTGIGL
PtHK2 ATVSHEIRTPMNGVLGMGDPGRFRQIITNLVGNSIKVTVEDTGVGIPIFTPFMQGTGIGL
PpHK2 ATVSHEIRTPMNGVLGMGDPGRLRQIITNLVGNKLLVTVEDTGVGIPIFTPFMQGTGIGL
AHK3 ATVSHEIRTPMNGVLGMGDPGRFRQILTNLMGNSIKVSVEDTGVGIPIFTPFMQGTGIGL
PtHK3a ATVSHEIRTPMNGVLGMGDPGRFRQIITNLMGNSIKVSVEDTGEGIPVFTPFMQGTGIGL
PtHK3b ATVSHEIRTPMNGVLGMGDPGRFRQIITNLMGNSIKVSVEDTGEGIPVFTPFMQGTGIGL
PpHK3 ATVSHEIRTPMNGVLGMGDPGRFRQIITNLMGNSIKVSVEDTGVGIPVFTPFMQGTGIGL
AtCRE1 ATVSHEIRTPMNGILGMGDSGRFRQIIINLVGNSVKVSIEDTGIGIPVFMPFMQGTGIGL
PtCRE1a ATVSHEIRTPMNGVLGMGDPGRFRQIITNLVGNSVKVCVEDTGIGIPVFMPFVQGTGIGL
PtCRE1b ATVSHEIRTPMNGILGMGDPGRFRQIITNLVGNSVKVCVEDTGIGIPVFMPFVQGTGIGL
PpCRE1 ATVSHEIRTPMNGILGMGDPGRFRQIITNLVGNSIKVSVEDTGIGIPVFMPFMQGTGIGL
AtCKI1 ANASHDIRGALAGMKGLGDSGRLKQILNNLVSNAVKFEVDDTGKGIPVFENYVQGTGLGL
PtCKI1a ASASHDIRAALAGITGLGDRVKLKQVLCNLLSNAVKFEVNDTGKGIPVFENFVQGTGLGL
PtCKI1b AGANHDVRNSLAAVRASGDRLKLKQILCNLVNNAIKFEVDDTGKGIPLFEDYVQGTGLGL
PtCKI1c ATASHDIRAALAGITGLGDRGKLKQVLCNLLSNAVKFEVNDTGKGIPVFENFVQGTGLGL
PpCKI1a SRANHDVRTSLAAITVRGDRGKLKQIMCNLLSNAVKFEVDDTGEGIPVFENFVQGSGLGL
PpCKI1b ATVSVLYYYSLCTCILFGDMGRLKQILCNLISNAVKFEVDDTGKGIPVFENYVQGTSLGL
PpCKI1c VSASHDVRAALTGITGLGDGGRLKQILCNLLSNAVKFEVDDTGKGIPVFENYVQGTGLGL
AtCKI2 ATMSHEIRSPLSGVVGMGDVLRIRQILTNLISNAIKCDVWDTGIGIPLFKKYMQGTGLGL
AtPHYA AYIKRQIRNPLSGIMFTGDSIRLQQVLADFMLMAVND-VSEEGLLV-KLMNKSSFIITAE
AtPHYB AYICQVIKNPLSGMRFAGDQIRIQQLLAEFLLSIIRWTSPE-GLGILKLMNRSYFLIIL-
AtPHYC AYLRHEVKDPEKAISFLGDNLRLQQILSETLLSSIRGTSRE-GLGLVKLMERSAFVILTE
AtPHYD AYIFQVIKNPLSGLRFTGDQIRLQQVLAEFLLSIVRWTSPE-GLGILKLMNRSYFLIVI-
AtPHYE TYVRQEIKNPLNGIRFAGDRVKLQLILADLLRNIVNWVTPD-GLGLLEQMNRCFFQVDLQ
AtETR1 AVMNHEMRTPMHAIIALGDEKRLMQIILNIVGNAVK--VKDSGAGINIFTKFAQGSGLGL
AtETR2 KTMSEGMRRPMHSILGLGDERRVFQVILHIVGSLVKVENDDSSSQS-—FSDQEVGQDLSF
AtERS1 AVMNHEMRTPMHAIISLGDEKRLMQTILNIMGNAVKQ-VKDTGCGIHLFTKFVQGGGLGL
AtERS2 QMMSDAMRCPVRSILGLGDDRKVFQAILHMLGVLMNAEGEESSSSNLEEEEENPSL----
AtEIN4 KVMSHGMRRPMHTILGLGDEKRTFQLVMYMLGYILDIQNPPLDGAHIP-NRRKEGLSLGM

AHK1 CIVRRILLAEDTPVLQRVDLILMDCQMPKMDGYEATKPIVALTGMDAYLTKPI
AHK2 SISKQILVVDDNLVNRRVDACFMDLQMPEMDGFEATRPILAMTGMDGYVSKPF
PtHK2 SISKQILVVDDNLVNRRVDACFMDFQMPEMDGFEATRPILAMTGMDGYVSKPF
PpHK2 SISKKILVVDDNAVNRRVEACFMDLQMPEMDGFEATRPILAMTGMDDYVSKPF
AHK3 SISKKILIVDDNNVNLRVDACFMDIQMPEMDGFEATRPVLAMTGMDGYVSKPF
PtHK3a SISKKILIVDDNKVNLIVDACFMDIQMPEMDGFEATRPILAMTGMDGYVSKPF
PtHK3b SISKKMLIVDDNKVNLMVDACFMDIQMPEMDGFEATRPILAMTGMDGYVSKPF
PpHK3 SISRKILIIDDNNVNLRVDACFMDIQMPEMDGFEATRPILAMTGMDGYVSKPF
AtCRE1 SISKKILVVDDNIVNRRVDACFMDIQMPQMDGFEATRPILAMTGMDGYVSKPF
PtCRE1a SISKRILVVDDNRVNRRVDACFMDIQMPEMDGFEATRPILAMTGMDGYVSKPF
PtCRE1b SISKKILVVDDNRVNRRVDACFMDIQMPEMDGFEATRPILAMTGMDGYVSKPF
PpCRE1 SISKKILVVDDNRVNRRVDACFMDIQMPEMDGFEATRPILAMTGMDGYVSKPF
AtCKI1 GIVQRVLVVDDNFISRKVDYIFMDCQMPEMDGYEATRPIIAVSGMDAFLDKSL
PtCKI1a GIVQKLLVVDDNEISRKVDYILMDCEMPKMDGCEATRPILAFSGTDGRVNKKI
PtCKI1b GIVQNVLVVEDS-LLQRLDIIFMDCEMPVMNGFEATRPIIALTGMDFHLTKPL
PtCKI1c GIVQKFLVADDNEISRRVVLISCIQQMPKMDGCEATRPILAFSGTDGRVNKKI
PpCKI1a GVVQSVLLVEDSYLKKLGDYIFMDCEMPIMNGFEATRPIIALTGMDVHLTKPL
PpCKI1b GIVVALLVIDATAGPFKMNYIQMDCEMPEMDGFEATMPIIPLTGHAPGEERKK
PpCKI1c GIVQTLLVIDTTATHRKHDYILMDCQMPIMDGFEAARPIIALTGMDHHLTKPL
AtCKI2 AICKKILLVEDNKINIMVDLVLMDVCMPVLDGLKATRPIIAMTGMDSFISKPV
AtPHYA -----------------------------------------------------
AtPHYB -----------------------------------------------------
AtPHYC -----------------------------------------------------
AtPHYD -----------------------------------------------------
AtPHYE -----------------------------------------------------
AtETR1 AISKKVLVMDENGVSRMV-VVFMDVCMPGVENYQIALLLVALSGLDGVLLKPV
AtETR2 GVCKQVLLVDTND-SNRA-VVVLDLQMAEMDGYEVAMLIVATTGINGVVRKPV
AtERS1 ALCK--------------------------------- -------------
AtERS2 -----------------------------------------------------
AtEIN4 CRRITLADDDD--VNRTV-VVILDLQMPEMDGFEVAMLIIALTGMNGMIQKPV
