# Supplementary material for: Characterization of cytokinin signaling and homeostasis gene families in two hardwood tree species: Populus trichocarpa and Prunus persica
Source: BMC Genomics. 2013 Dec 16;14:885. doi: 10.1186/1471-2164-14-885 (PMC3866579; doi:10.1186/1471-2164-14-885)
Supplement: Additional file 8: Figure S5 — Alignment of Populus (Pt), Prunus (Pp) and Arabidopsis histidine phosphotransfer proteins (HPts). The consensus HQxKGSSxS motif, containing the phospho-accepting histidine residue (H), is marked above the alignment. Altogether four Populus (PtHPt6a, PtHP6b, PHP4b, and PtHP-like), and two Prunus (PpHP6 and PpHP-like) HPts lack the conserved histidine residue. [file 1471-2164-14-885-S8.docx]

Supplementary Figure 5

AHP1 1 ---MDLVQKQKSLQDYTKSLFLEGILDSQFLQLQQLQDESNPDFVSQVVTLFFQDSDRIL
PtHP1b 1 ---MEVVQMQRAWVEYTKSLFREGFLDAQFQQLQLLQDESNPDFVAEVVSLFFEDSERLL
PtHP1a 1 ---MEVGQMQRAWVEYTKSLFREGFLDAQFQQLQLLQDESNPDFVAEVVSLFFEDSERLL
PpHP1 1 -----VGQMQRQWVDYTKSLFLEGFLDGQFLQLQQLQDESNPDFVVEVVSLFFEDSEKLL
AHP2 1 -MDALIAQLQRQFRDYTISLYQQGFLDDQFTELKKLQDDGSPDFVSEVLSLFFEDCVKLI
AHP3 1 -MDTLIAQLQRRFCDFTISLYHQGFLDDQFTELKKLQDECSPDFVAEVVTLFFEDCEKLI
PtHP7c 1 --MDVVSQLQRQLADFLAPLYREGFVDDQFTQLQKLQDESSPDFVMEVVSLFFEDCEKLV
PtHP8b 1 --MDSVVQLQRQLVDYTGQLFNEGFLDDQFNQLQQLQDESNPDFVVELVTLFFEDSEKLI
PtHP7a 1 --MDLLNQLQRQYTDFTTLLYHEGFVDDQFTQLQKLQDESSPDFVVEVVSLFFEDCEKLV
PpHP7 1 --MDAVTQWQKQWFDYTQSLRREGFLDDQFAQLKKLQDESSPDFVVEVVSLFFQDSEKLL
AHP4 1 --------------MTNIGKCMQGYLDEQFMELEELQDDANPNFVEEVSALYFKDSARLI
PtHP4a 1 ---MERNQLRRQVALTRQSLFDQGFLDEQFIQLEELQDDANPNFVEEVVSLHYRDSARLI
PtHP4c 1 ---MDRKQLQHQVASTRRSLFDQGYLDDQFIQLEHLQDEANPNFVEEVVRLFYSDSVRLI
PpHP4c 1 ---MDKNQSRRQVRLMRQSLFDQGFLDEQFIQLEELQDDSNPNFVEEIATSYYRDSYRSL
PpHP4d 1 ---MDKNQ--RQVRLMRQSLFDQGFLDEQFIQLEELQDDANPNFVEEIATSYYQDSSRSL
AHP5 1 MNTIVVAQLQRQFQDYIVSLYQQGFLDNQFSELRKLQDEGTPDFVAEVVSLFFDDCSKLI
AHP6 1 MLGLGVDRLQADINRLLASLFHQGVLDEQFLQLQQLQDETSPNFVYDVINIYFDESEKLL
PtHP6a 1 MLGWGVDRLRADMSRLLAILFHQGVLDEQFLQLQQLQDESSPNFVSEVVTIYFHESEKLL
PtHP6b 1 MLGWGVDRLRADMNRLLAMLFHQGVLDEQFLQLQQLEDESSPNFVSEVVNIYFHESEKLL
PpHP6 1 MLGLGADRLRADMNRLLAMLFHQGVLDEQFLQLQQLQDESSPNFVSEVVNIYFHESEKLL
At4g04402 1 ----LGMDLN-WIGYFVGSSGFLGFLDDQFTELKKLQDDGSPDFVAEVLSLFFEDCVKLI
PtHP8a 1 --MDSVVQLQRQLVDYSAQLFNEGFLDDQFNQLQQLQDESNPDFVVEVVTLFFEDSERLL
PtHP7b 1 --MGVFG-------------FEKGFVDDQFTQLQKLQDESSPGFVVEVVSLFFEDCEKLV
PpHP8 1 TMADVVSHLQKQLVDYTASLFDEAFLDEQFNQLQQLQDENNPDFVVEVVSLFFKDSERLM
PtHP4b 1 --MAS-TPLRQQLSTMRQSFFDEGLLDGQVSYLETLENEDDPDFIENIFTLFLRDSSKYI
PtHP4d 1 ---MGGPNIWEEQRNFVKNLHEQGILDSRFDEILDLPRE-NPQFVIDLVTKFCSDAENSI
PpHP4a 1 -------MLRQQVASMRQSLFDEEILDTQFMQMEQLEDVDNPNFAEEVMTLYFRDSTKLI
PpHP4b 1 ---MDRNQLQRQVALKRSSLFEQ-----QFVQLEDLEDDANPNFVEEIVTLFYKDSSRLF
PpHP-like 1 ---MALPILKGLLRGYVQSLFNEGIVNNHFSQIQTLKSDADPDCAVRLINIYLLDVERML
PtHP-like 2 TGLAALEALTEKLYDFIQSMEDDGIVDHHFKDCYNLKEANGPFLFIELLPTYISDSETTL

AHP1 NDLSLSLDQQ-VVDFKKVDPHVHQLKGSSSSIGAQRVKNACVVFRSFCEQQNVEACHRCL
PtHP1b SDLTFALEQQ-SVDFKKVDAHVHQLKGSSSSIGAQRVKNDCIAFRSFCEEQNIEGCQRCL
PtHP1a TDLTSALEQQ-NIDFKKVDAHVHQFKGSSSSIGALRVKNDCIAFRNFCEEQNIEGCLRCL
PpHP1 NDLTRALEQP-SVDFKRVDAHVHQFKGSSSSIGAQRVKNACIAFRNFCEEQNTEGCVRCV
AHP2 SNMARALDTTGTVDFSQVGASVHQLKGSSSSVGAKRVKTLCVSFKECCEAKNYEGCVRCL
AHP3 SNMARALDQTGNVDFKLVGSSVHQLKGSSSSVGAKRVKGLCVTLKECCDSQNYEGCVRCL
PtHP7c NNMAKALEQQ-VVDFKQVDSHVHQLKGSSSSIGAARIKNVCIAFKTFCEAQNREGCLRCL
PtHP8b NELAKDLEQQ-SIDYRKIDAHVHQLKGSSSSIGVQRVQRVCIAFRNYCEERNIEGCQKCL
PtHP7a NNMAKALEQQ-DVDFKQVDSHVHQLKGSSSSIGAARIKNVCIAFKTFCEAQNREGCLRCL
PpHP7 NNMGRALEQN-VVNFKQVDAYVHQFKGSSAWIGASRLKNVCINFRNSCEAQNLEGCLRCL
AHP4 NNIDQALERGS-FDFNRLDSYMHQFKGSSTSIGASKVKAECTTFREYCRAGNAEGCLRTF
PtHP4a SNIEKALEKNP-LDFNKLDGYMHQFKGSSSSIGAKKVKAECTLFREYCKAGNGEGCMRTF
PtHP4c QNIEQAMINKPNIDFGKLDDYMHQFKGSSSSIGAKKVMKECSKFREYCYAGNIEGCIKTF
PpHP4c QAIELALEKTP-RDFSKLDSYMHQFKGSSSSIGAKKVKAECQQFREYCNAGNGEGCMRTF
PpHP4d QTIELALEKTP-HDFNKLDGYMHQFKGSSSSIGAKKVKAECQQFREYCKAGNGEGCMRTF
AHP5 NTMSISLERPDNVDFKQVDSGVHQLKGSSSSVGARRVKNVCISFKECCDVQNREGCLRCL
AHP6 RNLRLLLMDREFSDYKKIGLHLNQLVGSSSSIGARRVRNVCVAFRSASELSNRPGCLRGL
PtHP6a RNLRGLLLDREFSDYKKMGIHLNQFMGSSSSIGAKRVRNVCVAFRAASEQNNRAGCLRAL
PtHP6b RNLRGLLMDREFSDYKKMGTHLNQLIGSSSSIGAKRVRNVCVSFRAASEQNSRAGCLRAL
PpHP6 KNLRELLMDREFSDYKKMGTHLNQFMGSSSSIGAKRIRNVCVAFRAASEHNNRAGCLRAL
At4g04402 SNMARALDKTGTVDFTLVFDTVLVRRGSKLCVLASRIAKLRITKGKLVQHRLRTHRLRCL
PtHP8a DELAKALEQQ-SVDYRKIDAHVHQLKGSSSSIGAQRVQKVCIGFRNFCEERNIEGCQKCL
PtHP7b NNMAKALEQQ-IVDFKQVDSHVHQLKGSSSSIGAARIQNVCIAFKTYCEGQNRDGCVRCL
PpHP8 DELNKALDQQ-TVDFKLVDKNIHQLKGSSSSIGVQRFQRACIAFRDCSEEQNVEGCLKSL
PtHP4b ASIEKALETAPDDFTFVLERMMYRLKGSSASIGATKINDETNKLRGFCHEGDLESAKASL
PtHP4d AALIRYHNEP-DINYPKVIDRAHQIKGASSCIGGHRMELRY-----ACEDKDKDRCFAAF
PpHP4a ATVEQALE-KPPYDVNKLDKSLHQLKGSSASVGANKVWIETNQMRESIKAGDLEGTKAQL
PpHP4b QKIEQTMQSRP-IDFGKLDDYMHQFKGSSSSIGAIKVKNECSQFKEFCLAGNAEGCFWAY
PpHP-like SELTCLSDLP-DVDFSKLATLARSIEEKSSLVGAEHVRSACADLIQACERMQKQNFLRAL
PtHP-like EEMTTELDQP-LVDFKHLEQLCIKLKGGTSCLGACRVATSCGEFRQAAIARNKDDCLLRL
 **HQxKGSSxS**

AHP1 QQVKQEYYLVKNRLETLFKLE--QQIVASGGMIPAVELG
PtHP1b QQVKQDYCLVKSKLEALIRLE--QQIVAAGGSIPMEELS
PtHP1a QQLKQDYYLVKSKLEALIRLE--QQIVAACGTIPMEELS
PpHP1 QQVKQEYYLVKNKLETLFAME--QQIVAAGGSIPILELS
AHP2 QQVDIEYKALKTKLQDMFNLE--KQIIQAGGIVPQVDIN
AHP3 QQVDIEYKTLKAKLQDLFNLE--QQIVQAGGRIPQVDI-
PtHP7c QQVNHEYTQLKANLQTLFTLE--RQIVAAGGSVPAMQ--
PtHP8b QQVKNEYSLVKTKLETLFKLE--QQVLAAGGSIPWPM--
PtHP7a QQVNHEHTQLKTNLQSLFTLE--RQIVANGGSIPVMQ--
PpHP7 QQVQQESSALKSKLEYLFMLE--QQIVAAGGSIPIME--
AHP4 QQLKKEHSTLRKKLEHYFQASQ-----------------
PtHP4a QQIKKEYATLKRKLETYFQLARQAGPA------------
PtHP4c QLLKQEHATLRRKLETYFQLVKQAGLA------------
PpHP4c QALKKEHATLKKRLEAYFQMARQAGPIEA----------
PpHP4d QALKREHATLKKRLEAYFQLARQAGPIET----------
AHP5 QQVDYEYKMLKTKLQDLFNLE--KQILQAGGTIPQVDIN
AHP6 EVVEHEYHYLKNMMHELFQLE-QQRILAAGVRYPM----
PtHP6a ELLEHEYCYLKNKLHELFQME-QQRVLAAGVRYPLHQQH
PtHP6b ELLEHEYCYLKNKLHELFHMD-QLRVLAAGVTYPHRSIE
PpHP6 EVLEHEYCYLKNKLHELFQIE-QQRVLAAGVRYPM----
At4g04402 QQVDIEYKTLTTKLQDMFNLE--KQIIQAGGRVPQVDIN
PtHP8a QQVRHEYSLVKTKLETLFKLE--QKILAAGGSIPWPM--
PtHP7b QQVNHECIQLKNNLQALFKLE--QQIVAAGGSIPVMQ--
PpHP8 QHVKHEYFLVKNKLETLFNLQ--KQLLAAGGPLP-----
PtHP4b QKLKAEHANFKQKLAVYVGIMKQVKLSEC----------
PtHP4d CKTKDEYQILKEKFNIILQVML-----------------
PpHP4a QLIKLAHETLRGKVEPYFHLVRQ----------------
PpHP4b QRVKQEHHTLRIQLESYFQSAEKLVMI------------
PpHP-like GWIKNEFAHTRNKLDSFVQMERRIFRVEG----------
PtHP-like EVIKGDFFILHNKLEAFLELERRIV--------------
