# Supplementary material for: Characterization of cytokinin signaling and homeostasis gene families in two hardwood tree species: Populus trichocarpa and Prunus persica
Source: BMC Genomics. 2013 Dec 16;14:885. doi: 10.1186/1471-2164-14-885 (PMC3866579; doi:10.1186/1471-2164-14-885)
Supplement: Additional file 9: Figure S6 — Alignment of Populus (Pt), Prunus (Pp) and Arabidopsis response regulators (RRs). [file 1471-2164-14-885-S9.docx]

Supplementary Figure 6

APRR8 ---------------------MIRKCGYKVVATTRADDLPLIINNKDKKIDLVLAEFRLI
APRR3 VRSLKVLLVENDDSTRHIVTALLKNCSYEVTAVPDVLEAWRILEDEKSCIDLVLTEVDMP
APRR7 VRTIRVLLVENDDCTRYIVTALLRNCSYEVVEASNGIQAWKVLEDLNNHIDIVLTEVIMP
APRR5 KIALRVLLVEADDSTRQIIAALLRKCSYRVAAVPDGLKAWEMLKGKPESVDLILTEVDLP
APRR9 KTVLRVLLVESDYSTRQIITALLRKCCYKVVAVSDGLAAWEVLKEKSHNIDLILTELDLP
APRR1 RSRVRILLCDNDSTSLGEVFTLLSECSYQVTAVKSARQVIDALNAEGPDIDIILAEIDLP
ARR1 PSGLRVLVVDDDPTCLMILERMLRTCLYEVTKCNRAEMALSLLRKNKHGFDIVISDVHMP
ARR2 PANLRVLVVDDDPTCLMILERMLMTCLYRVTKCNRAESALSLLRKNKNGFDIVISDVHMP
PtRR13 PAGLRVLVVDDDPTCLVILEKMLRTCLYEVTKCNRAEIALSLLRENRNGYDIVISDVHMP
PtRR24 PAGLRVLVVDDDPTCLVILEKMLRTCLYEVTKCNRAEIALSLLRENRNGYDIVISDVHMP
PpRR1 PAGLRVLVVDDDPTCLMILEKMLRTCLYEVTKCNRAEIALKLLRENKNGFDIVISDVHMP
PtRR15 PAGLRVLVVDDDITCLRLLEKMLRRCLYHVTTCSQATAALKLLRERKGCFDVVLSDVHMP
PtRR16 PAGLRVLVVDDDITCLRLLEKMLCRCLYNVTTCSQATAALKLLRERKGCFDVVLSDVHMP
PpRR7 PAGLRVLVVDDDTTCLRILEVLLGRCAYQVTACSQATVALNLLRERKGCFDVVLSDVHMP
ARR14 PSGLRILVVDDDTSCLFILEKMLLRLMYQVTICSQADVALTILRERKDSFDLVLSDVHMP
PtRR14 PAGLRVLVVDDDPTWLKILEKMLKKCSYEVTTCGLARDALNLLRERKGGYDIVISDVYMP
PtRR21 PAGLRVLVVDDDPTWLKILEKMLKRCSYEVTTCGLARDALNLLRERKGGYDIVISDVYMP
PpRR4 PAGLRVLVVDDDPTWLKILEKMLKKCSYEVTTCGLARDALNLLREKKDGYDIVVSDVNMP
ARR11 PVGLRVLVVDDDPTWLKILEKMLKKCSYEVTTCGLAREALRLLRERKDGYDIVISDVNMP
ARR10 PVGMRVLAVDDDQTCLRILQTLLQRCQYHVTTTNQAQTALELLRENKNKFDLVISDVDMP
ARR12 PVGMRVLAVDDDQTCLKILESLLRHCQYHVTTTNQAQKALELLRENKNKFDLVISDVDMP
PtRR22 PIGMRVLAVDDDPTCLLLLETLLRRCQYNVTTTSQAITALRMLRENKNKFDLVISDVHMP
PtRR23 PIGMRVLAVDDDPTCLLLLETLLRRCQYTVTTTSQAITALSMLRENKNKFDLVISDVHMP
PpRR6 PIGMRVLAVDDDPICLKLLDALLRRCKYHVTTTSQAIMALKLLRENKNKFDLVISDVHMP
PtRR18 PVGMRILAVDDDPICLKVLENLLRKCQYEVTTTNQAVTALEMLRENRNKYDLVISDVNMP
PtRR19 PVGMRVLAVDDDPICLKVLENLLRKCQYEVTTTNQAVTALEMLRENRNKYDLVISDVNMP
PpRR5 PVGMRVLAVDDNPICLRLLEGLLQNCQYQVTTTTQAVEALLMLRESRNRFDLVITDVSMP
ARR18 ---MRVLAVDDNPTCLRKLEELLLRCKYHVTKTMESRKALEMLRENSNMFDLVISDVEMP
PtRR25 PADLRVLAIDANVVCLKYLVAILQKCQYRVTSTTLAAEALKMLRENKNDYHVVITDVKRL
APRR4 PEGLRVLVFDEDPSYLLILERHLQKFQYQVTICNEVNKAMHTLRNHRNRFDLAMIQVNNA
ARR19 PGNTNVLVVDTNFTTLLNMKQIMKQYAYQVSIETDAEKALAFLTSCKHEINIVIWDFHMP
ARR20 ---------------------------------------MAFLMKNKHEIDLVIWDFHMP
APRR2 LLLLNGCDSDGDGSSAAETRSELESMDYIVTTFTDETEALSAVVKNPESFHIAIVEVNMS
APRR6 ADDISILLIDHDTASIASLTSMLQQFSKRVMSVDVASKALSMIEKQKKEIGLIIANIEMP
ARR13 VLKINVMVVDDNRVFLDIWSRMLEKSKYRVIAVDYPKKALSTLKNQRDNIDLIITDYYMP
ARR21 VLKINVMVVDDDHVFLDIMSRMLQHSKYRVIAVDDPKKALSTLKIQRDNIDLIITDYYMP
ARR23 ------------------------------MTAKDSSEPVTTLT-QFNNIDVVITDYHMP
PtRR17 LPKIHILIVDDDSTSLSVVSATLKTVSYKVVTVKKPFDALSILRLKKGLFDLVVSDLHMP
PtRR20 LLKIKILVVDDDSTSLSIVSAMLKTCSYKVVTVKNPFDALSTLRLKKGLFDLVVTDLHMP
PpRR10 --MINILVVDDDATTLAIVSAMLKTWSYQVVSVRNPIDALATLRARKGIFDLVVTDLHMP
ARR3 SDQVHVLAVDDSLVDRIVIERLLRITSCKVTAVDSGWRALEFLGLDDDKAALIITDYCMP
ARR4 LDEVHVLAVDDSLVDRIVIERLLRITSCKVTAVDSGWRALEFLGLDNEKASLIITDYCMP
PtRR1 EEGVHVLAVDDSLVDRKVIERLLKISSCKVTAVDSGWRALKLLGLLD--EELIITDYCMP
PtRR2 EEEVHVLAVDDSFVDRKVIERLLKISSCKVTAVDSGWGALKLLGLLDDEEELIITDYCMP
ARR5 PKLLHVLAVDDSMVDRKFIERLLRVSSCKVTVVDSATRALQYLGLDGENNSLIMTDYSMP
ARR6 PDPLHVLAVDDSHVDRKFIERLLRVSSCKVTVVDSATRALQYLGLDVEEKSLIMTDYSMP
ARR7 PE-LHVLAVDDSIVDRKVIERLLRISSCKVTTVESGTRALQYLGLDGGKGALIVTDYSMP
ARR15 PE-LHVLAVDDSFVDRKVIERLLKISACKVTTVESGTRALQYLGLDGDNGSLIVTDYSMP
PtRR10 SEELHVLAVDDSFVDRKVIERLLKISSCKVTVVESGSRALQYLGLDGEKSSLIMTDYSMP
ARR8 ESKFHVLAVDDSLFDRKMIERLLQKSSCQVTTVDSGSKALEFLGLR--VDDLIITDYCMP
ARR9 ESQFHVLAVDDSLFDRKLIERLLQKSSCQVTTVDSGSKALEFLGLRQSTDSLIITDYCMP
PtRR4 ETQFHVLAVDDSLIDRKLIERLLKTSSYQVTAVDSGSKALEFLGLSGDDEQMIITDYCMP
PtRR5 ETQFHVLAVDDCLIDRKLIERLLKTSSYQVTAVDSGSKALEFLGLNGENELMIITDYCMP
PtRR6 HSQFHVLAVDDSMIDRKLIERLLKTSSYQVTAVDSGSKALKFLGLHEEDDHLIITDYCMP
PtRR7 LSQFHVLAVDDSLIDRKLIERLLKTSSYQVTTVDSGSKALKFLGLQE-DEQLIITDYCMP
PpRR3 DSQFHVLAVDDSLIDRKLIERLLKTSSYQVTTVDSGSKALEFLGLYE-DDQLVITDYCMP
PtRR3 EKHFHVLAVDDSFIDRKLLERLLKVSSYQVTFVDSGDKALEYLGLLDSID-LIMTDYCMP
PpRR2 EQHFHVLAVDDSLLDRKLLERLLRGSSYQVTCVESGDEALKYLGLVDDLNQLIMTDYCMP
ARR16 DEELHVLAVDDNLIDRKLVERLLKISCCKVTTAENALRALEYLGLGDQNQHLIITDYCMP
ARR17 EEELHVLAVDDNLIDRKLVERILKISSCKVTTAENGLRALEYLGLGDPQQTLIITDYCMP
PtRR8 TEEPHVLAVDDSLVDRKLVERLLKNSSCKVTTAENGLRALEYLGLGDEKRTLIITDYCMP
PpRR8 VEQPHVLAVDDSIIDRKLIEKLLKNLSCKVTTAENGLRALEFLGLGDHQNSLVITDYCMP
PtRR9 DEKLHVLAVDDGLIDRKAIERLLINSEYKVTTAENKKKAIEYLGLADGHHTLIITDYCMR
PtRR11 DEKPHVLAVDDSLIDRKVIERLLINSTCRVTTAENGKRALEYLGLADGQHPMIITDYSMP
PpRR9 DEKPHVLAVDDSFVDRRIIEKLLTNSACKVTTAENAQGALELLGLADGQQNLIITDYSMP
PtRR29 GYNFSVLIVDDDLTVRDTNRRLMMSVETQFQEAKNGKEAVYLHLAG-ASYDLILMENHMP
PtRR30 GNNFSVLIVDDDRTIRETNRRFMALAGTQFQEAKNGKEAVYLHLAG-ASFDLILMDNQMP
PtRR33 KNSFTVLVVDDDTVVRMVHRMLVTSLGLKVQEAKNGKEAVDLHING-ASFDLILMDMEMP
PtRR32 YCDLSVLIVDDDRAVRDSIRRAMMSYGAQVQEAKNGREAVYLHLGG-ASFDIILMDSQMP
PtRR26 GIKYCVLVVDDNCEDRESLRELLMSFKSQVQVAKNGKEAVYLHLAG-ASFDMIVMDDLMP
PtRR27 GNNFSVLVVHDSRDLRETRQRLLVLLGYQVSVAKNGKEAVYLHLAG-ASFDMIIMDDLMP
ARR22 -----VLIVDDDPLNRRLHEMIIKTIGGISQTAKNGEEAVILHRDGEASFDLILMDKEMP
PtRR28 GTKMTALVVDDDSINQTIHHRLLEQLGIENQVARNGKEAIDIHCSG-KKFDLILMDRDMP
PtRR31 -TKITALVVDDDRIIKTIHSKLLSKLGIENQVAANGEEAVDLHCSG-KKFDLIVMDRDMP

ARR24 ESKLTALVVDDSFVNQTIHQKLLNRLGIKNDVVTNGKEAVDVYCSG-GNYDLILMDMDMP

APRR8 EMNK-YELLEKIR---SICEIPVVV-SGAHVKDAIVECLCRGAKLCLEKPLMENDFKILW
APRR3 VHS-GTGLLSKIMSHKTLKNIPVIMMSSHDSMVLVFKCLSNGAVDFLVKPIRKNELKNLW
APRR7 YLS-GIGLLCKILNHKSRRNIPVIMMSSHDSMGLVFKCLSKGAVDFLVKPIRKNELKILW
APRR5 SIS-GYALLTLIMEHDICKNIPVIMMSTQDSVNTVYKCMLKGAADYLVKPLRRNELRNLW
APRR9 SIS-GFALLALVMEHEACKNIPVIMMSSQDSIKMVLKCMLRGAADYLIKPMRKNELKNLW
APRR1 MAK-GMKMLRYITRDKDLRRIPVIMMSRQDEVPVVVKCLKLGAADYLVKPLRTNELLNLW
ARR1 DMD-GFKLLEHVG---LEMDLPVIMMSADDSKSVVLKGVTHGAVDYLIKPVRMEALKNIW
ARR2 DMD-GFKLLEHVG---LEMDLPVIMMSADDSKSVVLKGVTHGAVDYLIKPVRIEALKNIW
PtRR13 DMD-GFKLLELIG---LEMDLPVIMMSADDGKNVVMKGVTHGACDYLIKPIRIEALKNIW
PtRR24 DMD-GFKLLELIG---LEMDLPVIMMSADDGKNVVMKGVTHGACDYLIKPIRIEALKNIW
PpRR1 DMD-GFKLLERVG---LEMDLPVIMMSADDGKSVVMKGVTHGACDYLIKPVRIEALKNIW
PtRR15 DMD-GFKLLELVG---LEMDLPVIMMSADGRTSAVMRGISHGACDYLIKPIREEELKNIW
PtRR16 DMD-GFKLLELVG---LEMDLPVIMMSADGRTSAVMRGIRHGACDYLIKPIREEELKNIW
PpRR7 DMD-GFKLLEHVG---LEMDLPVIMMSADGRTSAVMQGIKHGACDYLIKPIHEAELKNIW
ARR14 GMN-GYNLLQQVGL--LEMDLPVIMMSVDGRTTTVMTGINHGACDYLIKPIRPEELKNIW
PtRR14 DMD-GFKLLEQVG---LEMDLPVIVMSVDGETSRVMKGVQHGACDYLLKPIRMKELRNIW
PtRR21 DMD-GFKLLEHVG---LEMDLPVIMMSVDGETSRVMKGVQHGACDYLLKPIRMKELRNIW
PpRR4 DMD-GFKLLEHVG---LEMDLPVIMMSVDGETSRVMKGVQHGACDYLLKPIRMKELRNIW
ARR11 DMD-GFKLLEHVG---LELDLPVIMMSVDGETSRVMKGVQHGACDYLLKPIRMKELKIIW
ARR10 DMD-GFKLLELVG---LEMDLPVIMLSAHSDPKYVMKGVKHGACDYLLKPVRIEELKNIW
ARR12 DMD-GFKLLELVG---LEMDLPVIMLSAHSDPKYVMKGVTHGACDYLLKPVRIEELKNIW
PtRR22 DMD-GFKLLELVG---LEMDLPVIMLSANGDPKLVMKGITHGACYYLLKPVRIEELKTIW
PtRR23 DMD-GFKLLELVG---LEMDLPVIMLSANGDPKLVMKGITHGACDYLLKPVRIEELKNIW
PpRR5 DMD-GFKLLELVG---LEMDLPVIMLSANGDTKLVMKGITHGACDYLLKPVRIEQLKNIW
PtRR18 DMD-GFKLLELVG---LEMDLPVIMLSSHGDKEFVYKGVTHGAVDYLLKPVRMEELKNIW
PtRR19 DMD-GFKLLELVG---LEMDLPVIMLSSHGDKEFVFKGITHGAVDYLLKPVRLEELKNIW
PpRR6 DMD-GFKLLELLG---LEMDLPVIMLSGHSDKELVMKGISHGACDYLLKPVRKEELTNIW
ARR18 DTD-GFKLLEI-G---LEMDLPVIMLSAHSDYDSVMKGIIHGACDYLVKPVGLKELQNIW
PtRR25 DMD-GFKLLEIIG---LEMDLPVILVSAEDSQSSIMKGIRHGARDYLLKPVRIQEMQNIW
APRR4 EGDI-FRFLSEIG---SEMDLPIIIISEDDSVKSVKKWMINGAADYLIKPIRPEDLRIVF
ARR19 GID-GLQALKSIT---SKLDLPVVIMSDDNQTESVMKATFYGACDYVVKPVKEEVMANIW
ARR20 DIN-GLDALNIIG---KQMDLPVVIMSHEYKKETVMESIKYGACDFLVKPVSKEVIAVLW
APRR2 AESESFKFLEAAK-----DVLPTIMISTDHCITTTMKCIALGAVEFLQKPLSPEKLKNIW
APRR6 HIDS-HSFLNALL----LKDIPLILINPEIKTKEPSDLLTKRACFSLDKPISNDDIKNMW
ARR13 GMN-GLQLKKQITQ--EFGNLSVLVMSSD--PNKEEESLSCGAMGFIPKPIAPTDLPKIY
ARR21 GMN-GLQLKKQITQ--EFGNLPVLVMSSD--TNKEEESLSCGAMGFIPKPIHPTDLTKIY
ARR23 GLN-GVQLKKRIDE--EFGNLPVIDLYR----NIEHEELFRRALCFMHKPISRRDLNSVC
PtRR17 EMN-GMELQKQVEE--EFK-LPVIIMSSDESKNVISRSLEGGAAFYIVKPANKVDLKNVW
PtRR20 EMN-GMELQQQVDE--EFK-LPVIIMSSDDSEKVILRTLEGGAAFYIVKPINKDDLKNVW
PpRR10 QMN-GFELQKHVHE--EFK-LPVIMMSADDKESVILKSLEGGVVYYIVKPVSKDDIKNVW
ARR3 GMT-GYELLKKIKESTSFKEVPVVIMSSENVMTRIDRCLEEGAEDFLLKPVKLADVKRLR
ARR4 GMT-GYELLKKIKESSNFREVPVVIMSSENVLTRIDRCLEEGAQDFLLKPVKLADVKRLR
PtRR1 GMT-GYELLKKIKESTTFREIPVVIMSSENVVARIDRCLEEGAEDFIVKPVKLSDVKRIR
PtRR2 GMT-GYELLKKIKESSSFREIPVVIMSSENVMARIDRCLEEGAEEFIAKPVKLSDVKRLR
ARR5 GMT-GYELLKKIKESSAFREIPVVIMSSENILPRIDRCLEEGAEDFLLKPVKLADVKRLR
ARR6 GMT-GYELLKKIKESSAFREVPVVIMSSENILPRIDRCLEEGAEDFLLKPVKLSDVKRLR
ARR7 GLS-GYDLLKKIKESSAFREVPVVIMSSENILPRIQECLKEGAEEFLLKPVKLADVKRIK
ARR15 GLT-GYELLKKIKESSALREIPVVIMSSENIQPRIEQCMIEGAEEFLLKPVKLADVKRLK
PtRR10 GMT-GYELLKKIKESSAFREIPVVIMSSENILARIDRCLEEGAEEYILKPVKLSDVKRIK
ARR8 GMT-GYDLLKKVKESAAFRSIPVVIMSSENVPARISRCLEEGAEEFFLKPVKLADLTKLK
ARR9 GMT-GYDLLKKVKESSAFRDIPVVIMSSENVPARISRCLEEGAEEFFLKPVRLADLNKLK
PtRR4 GMT-GYDLLKKIKESKYFKDIPVVIMSSENVPSRINRCLEEGAEEFFLKPVQLSDVNKLR
PtRR5 GMT-GYDLLKKIKESKYFKDIPVVIMSSENVPSRINRCLKEGAEEFFLKPVQLSDVNKLR
PtRR6 GMT-GYDLLKKVKESSSLRDIPVVIMSSENVPSRITRCLEEGAEEFFLKPVRLADLNRLK
PtRR7 GMT-GYDLLKKVKESSSLRNIPVVIMSSENVPSRITRCLEEGAEEFFLKPVRLSDLNRLK
PpRR3 GMT-GYDLLKKIKESSSLRNIPVVIMSSENVPSRINRCLEEGAEEFFLKPVRLSDLSRLR
PtRR3 GMS-GYDLLKRVKGS-YWKDVPVVVMSSENIPSRIRMCLEEGAEEFLLKPLQLSDVEKLQ
PpRR2 GMS-GYDLLKRVKGS-SWKNVPVVVMSSENVPSRISMCLEGGAEEFLLKPLQLSDLKKLQ
ARR16 GMT-GFELLKKVKESSNLREVPVVIMSSENIPTRINKCLASGAQMFMQKPLKLADVEKLK
ARR17 GMT-GFELLKKVKESSNLKEVPVVILSSENIPTRINKCLASGAQMFMQKPLKLSDVEKLK
PtRR8 GMT-GYELLKKIKESSMLKEIPVVIMSSENIPTRINKCLEEGAQMFMLKPLKQSDVVKLR
PpRR8 GMT-GYELLKKIKESSLMKEVPVVIMSSEYVPNRINKCLEEGAQMFMLKPLRQSDIKKLR
PtRR9 GMT-GYELLKRIK------EIPVVVVSSENIPTRIKQCMEEGAQEFLLKPLQLSGVTKLS
PtRR11 GMT-GYELLKRIKESPTMKEIPVVVVSSENIPTRINQCMEGGAQEFLLKPLQLSDATKLR
PpRR9 GMT-GYELLKKIKESPTGKEIPVVVVSSEHIPTRIEKCLEEGAKEFLLKPLRQSDVNQLR
PtRR29 IMT-GIQATQRLRKMGVKSQIVGISS-----ESDQQAFIDAGLDNCIQKPLDIAKITTFL
PtRR30 IMT-GIQATQLLRKMGVKSRIVGVTS-----EPDRQAFIDAGLNNCIQKPLNPGKITEFL
PtRR33 IMN-GPTATRELRAMGVKSTIIGVTSCT-F-ESVHKDFMEAGLNHCVAKPLTIAQIASFL
PtRR32 VMK-GHEAVKKLRQMGVKSRIIGVSY-----QFEKPAFRGSSINKWIKKPLNLEKIAAIF
PtRR26 VMN-GIEATKQLFGMGVISFIVGVGD-----DTVKQAFIDAGIDQYIEKPLTPAKVADLF
PtRR27 FMD-GIEAINLLRRMGVESRIVGVTG-----EFKRLAFMDSGADSCIIKPLTLEKLAAVF
ARR22 ERD-GVSTTKKLREMKVTSMIVGVTSVADQ-EEERKAFMEAGLNHCLEKPLTKAKIFPLI
PtRR28 IMN-GIEATRKLRAMGIRSMIAGVSTRCV--KQEIQEFMEAGLDDYQEKPLTSAKIISIL
PtRR31 IMN-GIEATRELRALGIRSIIVGVSTRSL—-EQEIQEFMDAGLDDYQEKPLTSSKVISIL

ARR24 IMN-GIQATKRLREMGIESKIAGVTTRAN--EGEKKEFMEAGLNDFQEKPLTISKLLSIL
APRR8 QFTV--------------------------------------------------------
APRR3 QHVWR-------------------------------------------------------
APRR7 QHVWR-------------------------------------------------------
APRR5 QHVWR-------------------------------------------------------
APRR9 QHVWR-------------------------------------------------------
APRR1 THMWR-------------------------------------------------------
ARR1 QHVVRGE-EQGDDKDEDASNLKKPRVVWSVELHQQFVAAVNQLGVEKAVPKKILELMNVP
ARR2 QHVVRVD-DQGDDK-EDSSSLKKPRVVWSVELHQQFVAAVNQLGVDKAVPKKILEMMNVP
PtRR13 QHVVRDE-EEEADERDDTSTLKKPRVVWSVELHQQFVAAVHQLGIDKAVPKKILELMNVP
PtRR24 QHVVRDE-EEEADERDDTSTLKKPRVVWSVELHQQFVAAVHQLGIDKAVPKKILELMNVP
PpRR1 QHVVRDE-EEEADERDDSSTLKKPRVVWSVELHQQFVGAVNQLGIDKAVPKKILELMNVP
PtRR15 QHVIRDE-DDAELENDDPSASKKPRVVWSVELHQQFVSAVNHLGIDKAVPKRILELMNVP
PtRR16 QHVIREE-DDNELEIDDPSASKKPRVVWSVELHQQFVSAVNQLGIDKAVPKRILELMNVP
PpRR7 QHVVRED-DDGDMESDDPSTSKKPRVVWSVELHQQFVSAVNQLGLDKAVPKRILELMNVP
ARR14 QHVVRDD-NEDDLLLD-PGNSKKSRVVWSIELHQQFVNAVNKLGIDKAVPKRILELMNVP
PtRR14 QHVFRES-KHDEKDTGDSISTKKARVVWSVDLHQKFVKAVNQIGFDTVGPKKILDMMNVP
PtRR21 QHVFRES-KHDEKDIGDNTSAKKARVVWSVELHQKFVKAVNQIGFDKVGPKKILDLMNVP
PpRR4 QHVFRDN-KYDDKDFSDCSSAKKARVVWSVDLHQKFVKAVHQIGFDTVGPKKILDLMNVP
ARR11 QHVLRKL-LQDESDP-SSSSSKKARVVWSFELHHKFVNAVNQIGCDHAGPKKILDLMNVP
ARR10 QHVVREE-EERGNDNDDPTAQKKPRVLWTHELHNKFLAAVDHLGVERAVPKKILDLMNVD
ARR12 QHVVRED-EDR-DDNDDSCAQKKQRVVWTVELHKKFVAAVNQLGYEKAMPKKILDLMNVE
PtRR22 QHVIRDE-DEDEHENEDPTTQKKPRVVWSVELHRKFVAAVNQLGVDKAVPKKILDLMNVE
PtRR23 QHVIRDR-DENEHENEDPATQKKPRV-----LHRKFVAAVNQLGIDKAVPKKILDLMNVE
PpRR6 QHVIRDR-DEDGHDNDDPSTQKKPRVVWSLELHRKFVAAVNQLGIDKAVPKKILDLMNVE
PtRR18 QHVIREG-EDG-DDNEVSGNQKKPRVVWSVDLHQKFVAAVNQMGLDKAVPKKILDLMNVD
PtRR19 QHVIREG-EDG-NDNEESGNQKKPRVVWSVELHQKFVSAVNQLGLDKAVPKKILDLMNVD
PpRR5 QHVIREY-EDDEHENEDPSTQKKPRVVWSKELHGKFVAAVHQLGLERAVPKKILDLMNVQ
ARR18 HHVVKDG-EGSEQDGDGSGTRKKPRVVWSQELHQKFVSAVQQLGLDKAVPKKILDLMSIE
PtRR25 QHVVREK-EIIGNPNGDACSGKKPRVTWSSELHVKFVDCVEKLEAERVQPKRIREMMNVE
APRR4 KHLVKED-RHDHNDRACASSAKKRRVVWDEELHQNFLNAVDFLGLERAVPKKILDVMKVD
ARR19 QHIVRGNGIQNMEKKQGKKP-RKPRMTWTEELHQKFLEAIEIIGANPKVLVECLQEMRIE
ARR20 RHVYRGS-KNTCDHKEEKSPTKKPRMQWTPELHHKFEVAVEKMGSLETILKYMQEELNVQ
APRR2 QHVVHIN-KSSGIKNVSGNKTSRKKVDWTPELHKKFVQAVEQLGVDQAIPSRILELMKVG
APRR6 QHVFSAI-EIENKRKEWKKSVGRRKSLWNSE--RHFIAAISILGEEDFRPKSILEIMNDP
ARR13 QFALTGG-PSDDGESLSQPP-KKKKIWWTNPLQDLFLQAIQHIGYDKVVPKKILAIMNVP
ARR21 QFALSGG-PSDDGESMSQPA-KKKKIQWTDSLHDLFLQAIRHIGLDKAVPKKILAFMSVP
ARR23 QQALRQN-VTDDPDDWLRLR-EKPKLKWTKPLQHRFMSALKSLGVASKY-----------
PtRR17 HYAVAKS-TKDDQEVDSQPASKKPKVVWTNSLHNRFLLALNHIGLDKAVPKRILECMSVR
PtRR20 QYAVAKR-TKEDQEVDSQLAPKKPKVVWTNSLHSRFLQAINHIGLDKAVPKRILEFMSVP
PpRR10 QYAVAKR-HREDDNEERMVAPKKPKVVWTNSLHNQFLLAIRHIGLDKAVPKRILEFMNVP
ARR3 SYLTR-------------------------------------------------------
ARR4 SHLTK-------------------------------------------------------
PtRR1 D-YMA-------------------------------------------------------
PtRR2 DYYMA-------------------------------------------------------
ARR5 DSLMK-------------------------------------------------------
ARR6 DSLMK-------------------------------------------------------
ARR7 QLIMR-------------------------------------------------------
ARR15 ELIMR-------------------------------------------------------
PtRR10 DVIMG-------------------------------------------------------
ARR8 PHMMK-------------------------------------------------------
ARR9 PHMMK-------------------------------------------------------
PtRR4 PHLMK-------------------------------------------------------
PtRR5 PHLMK-------------------------------------------------------
PtRR6 PHMMK-------------------------------------------------------
PtRR7 PHMMK-------------------------------------------------------
PpRR3 PHIMK-------------------------------------------------------
PtRR3 THLLK-------------------------------------------------------
PpRR2 PYLLK-------------------------------------------------------
ARR16 CHLMN-------------------------------------------------------
ARR17 CHLLN-------------------------------------------------------
PtRR8 CNLMN-------------------------------------------------------
PpRR8 CHLMK-------------------------------------------------------
PtRR9 AI----------------------------------------------------------
PtRR11 CHIKK-------------------------------------------------------
PpRR9 CHLMK-------------------------------------------------------
PtRR29 SDPNK-------------------------------------------------------
PtRR30 TVSKK-------------------------------------------------------
PtRR33 LKSNN-------------------------------------------------------
PtRR32 C-----------------------------------------------------------
PtRR26 PDLSD-------------------------------------------------------
PtRR27 RDH---------------------------------------------------------
ARR22 SHLFD-------------------------------------------------------
PtRR28 HKIDH-------------------------------------------------------
PtRR31 HKINH-------------------------------------------------------

ARR24 HKLNF-------------------------------------------------------

APRR8 ----------------------------------------------
APRR3 ----------------------------------------------
APRR7 ----------------------------------------------
APRR5 ----------------------------------------------
APRR9 ----------------------------------------------
APRR1 ----------------------------------------------
ARR1 GLTRENVASHLQKYRIYLRRLGGVSQHQGNLNNS---FMTGQDASF
ARR2 GLTRENVASHLQKYRIYLRRLGGVSQHQGNMNHS---FMTGQDQSF
PtRR13 GLTRENVASHLQKYRLYLRRLSGVSQHQSGMGNS---FINPQEATY
PtRR24 GLTRENVASHLQKYRLYLRRLSGVSQHQSGMGNS---FINPQEATY
PpRR1 GLTRENVASHLQKYRLYLRRLSGVSQHPSNLNNS---FLSPQEASF
PtRR15 GLTRENVASHLQKFRLYLKRLSGVAQQGGISNP----FCGLLDSNV
PtRR16 GLTRENVASHLQKFRLYLKRLSGVAQQGGISNT----FCGPLDSNV
PpRR7 GLSRENVASHLQKFRLYLKRLA--QQQSGISNG----FCGPVESNG
ARR14 GLSRENVASHLQKFRLYLKRLSGEASQSNDS-----------ESTK
PtRR14 WLTRENVASHLQKYRLYLSRLQKENDFKNPAGGIKQSDSPLRDSAG
PtRR21 RLTRENVASHLQKYRLYLSRL-------------------------
PpRR4 WLTRENVASHLQKYRLYLSRLQKENDIKSSFGGTKHSDYSSKDLQG
ARR11 WLTRENVASHLQKYRLYLSRLEKGKELKCYSGGVKNADSSPKDVE-
ARR10 KLTRENVASHLQKFRVALKKVSDDAIQQANRAA----IDSHFMQMN
ARR12 KLTRENVASHLQKFRLYLKRISGVANQQAIMAN----SELHFMQMN
PtRR22 KLTRE------NKYRHYLKRISTVANQQANMVAALGSSDASYLQMN
PtRR23 KLTRE------NKYRLYLKRISTVANQQANMVAALGSSDASYLQMN
PpRR6 KLTRENVASHLQKYRLYLKRISRVANQQANMVAALGTSDSSYLQMT
PtRR18 GLTRENVASHLQKFRLYLKRLSSGGNQ-------------------
PtRR19 GLTRENVASHLQKFRLYLKRLSCGANQQPNMVAAFGAKDSSYLRMG
PpRR5 GLTRENVASHLQKYRLCLKRLSSTATQQASMVAAFGGKDSSYIRMG
ARR18 GLTRENVASHLQKYRLYLKKIDE-GQQQNMTPDAFGTRDSSYFQMA
PtRR25 GLSRENIASHLQKYRNLLKKHKDKMNQQDNAGTDLNRYGRISATKN
APRR4 YISRENVASHLQVTFLIYNIIVHFQQHFCFYS--------------
ARR19 GITRSNVASHLQKHRINLEENQIPQQTQ-GNGWATAYGTLAPSLQG
ARR20 GLTRNNVASHLQKYRQSSKKTCTPQEPQEDFVWGNAGPDVTLAASK
APRR2 TLTRHNVASHLQKFRQHRKNILPKDDHNHRWIQSR--NHRPNQRNY
APRR6 NLTHRQVGSHLQKYKAQIDQISYTLPRNESRSIDKT-FEYPSNYKY
ARR13 YLTRENVASHLQKYRLFVKRVVHQGR--FSMLSDR-GKDSMFRQTH
ARR21 YLTRENVASHLQKYRIFLRRVAEQGL--YSMLSDR-GIDSMFRQTH
ARR23 ----------------------------------------------
PtRR17 GLSRENIASHLQKYRIFLKKVAERGGCSSKNLSGRDDLRTNFAPSQ
PtRR20 GLSRENVASHLQKYRIFLKKVAERGTSSSKNLSGR-ALKSNFASSQ
PpRR10 GLTRENVASHLQKYRIFLKRVAEKAR-LSKCLSER-VFRSSFAFGH
ARR3 ----------------------------------------------
ARR4 ----------------------------------------------
PtRR1 ----------------------------------------------
PtRR2 ----------------------------------------------
ARR5 ----------------------------------------------
ARR6 ----------------------------------------------
ARR7 ----------------------------------------------
ARR15 ----------------------------------------------
PtRR10 ----------------------------------------------
ARR8 ----------------------------------------------
ARR9 ----------------------------------------------
PtRR4 ----------------------------------------------
PtRR5 ----------------------------------------------
PtRR6 ----------------------------------------------
PtRR7 ----------------------------------------------
PpRR3 ----------------------------------------------
PtRR3 ----------------------------------------------
PpRR2 ----------------------------------------------
ARR16 ----------------------------------------------
ARR17 ----------------------------------------------
PtRR8 ----------------------------------------------
PpRR8 ----------------------------------------------
PtRR9 ----------------------------------------------
PtRR11 ----------------------------------------------
PpRR9 ----------------------------------------------
PtRR29 ----------------------------------------------
PtRR30 ----------------------------------------------
PtRR33 ----------------------------------------------
PtRR32 ----------------------------------------------
PtRR26 ----------------------------------------------
PtRR27 ----------------------------------------------
ARR22 ----------------------------------------------
PtRR28 ----------------------------------------------
PtRR31 ----------------------------------------------

ARR24 ----------------------------------------------
